# Flexible Utility Function Approximation via Cubic Bezier Splines

**DOI:** 10.1007/s11336-020-09723-4

**Published:** 2020-09-26

**Authors:** Sangil Lee, Chris M. Glaze, Eric T. Bradlow, Joseph W. Kable

**Affiliations:** 1 Department of Psychology, School of Arts and Sciences, University of Pennsylvania, Philadelphia, PA USA; 2 Department of Marketing, Wharton School, University of Pennsylvania, Philadelphia, USA

**Keywords:** flexible modeling, heterogeneity, intertemporal choice, risky choice, generalized utility functions

## Abstract

In intertemporal and risky choice decisions, parametric utility models are widely used for predicting choice and measuring individuals’ impulsivity and risk aversion. However, parametric utility models cannot describe data deviating from their assumed functional form. We propose a novel method using cubic Bezier splines (CBS) to flexibly model smooth and monotonic utility functions that can be fit to any dataset. CBS shows higher descriptive and predictive accuracy over extant parametric models and can identify common yet novel patterns of behavior that are inconsistent with extant parametric models. Furthermore, CBS provides measures of impulsivity and risk aversion that do not depend on parametric model assumptions.

**Electronic supplementary material:**

The online version of this article (10.1007/s11336-020-09723-4) contains supplementary material, which is available to authorized users.

## Introduction

Intertemporal choices (ITCs) and risky choices (RCs) are heavily studied across many disciplines. ITCs are decisions regarding outcomes that occur at different times: for example, deciding between spending money now versus saving and investing that money for later, smoking now versus having better health later, or whether to pay an additional price for expedited shipping in order to receive a package earlier. RCs are decisions made regarding outcomes that occur probabilistically: for example, buying lottery tickets, investing in stock markets, or gambling. ITCs and RCs are studied both in basic and applied research. In basic research, researchers are interested in how people make ITCs or RCs and have generated many different proposals for the cognitive processes that underlie these choices. In applied research, researchers are often interested in how individual differences in ITC and RC relate to real-world behaviors such as pathological gambling, smoking, susceptibility to mental illness, drug and alcohol abuse, education level and financial status (Alessi and Petry 2003; Anderson and Mellor 2008; Brañas-Garza et al. 2007; Kirby et al. 1999; Krain et al. 2008; Lejuez et al. 2003, 2005; Lempert et al. 2019; Schepis et al. 2011; Shamosh and Gray 2008).

**Fig. 1:**
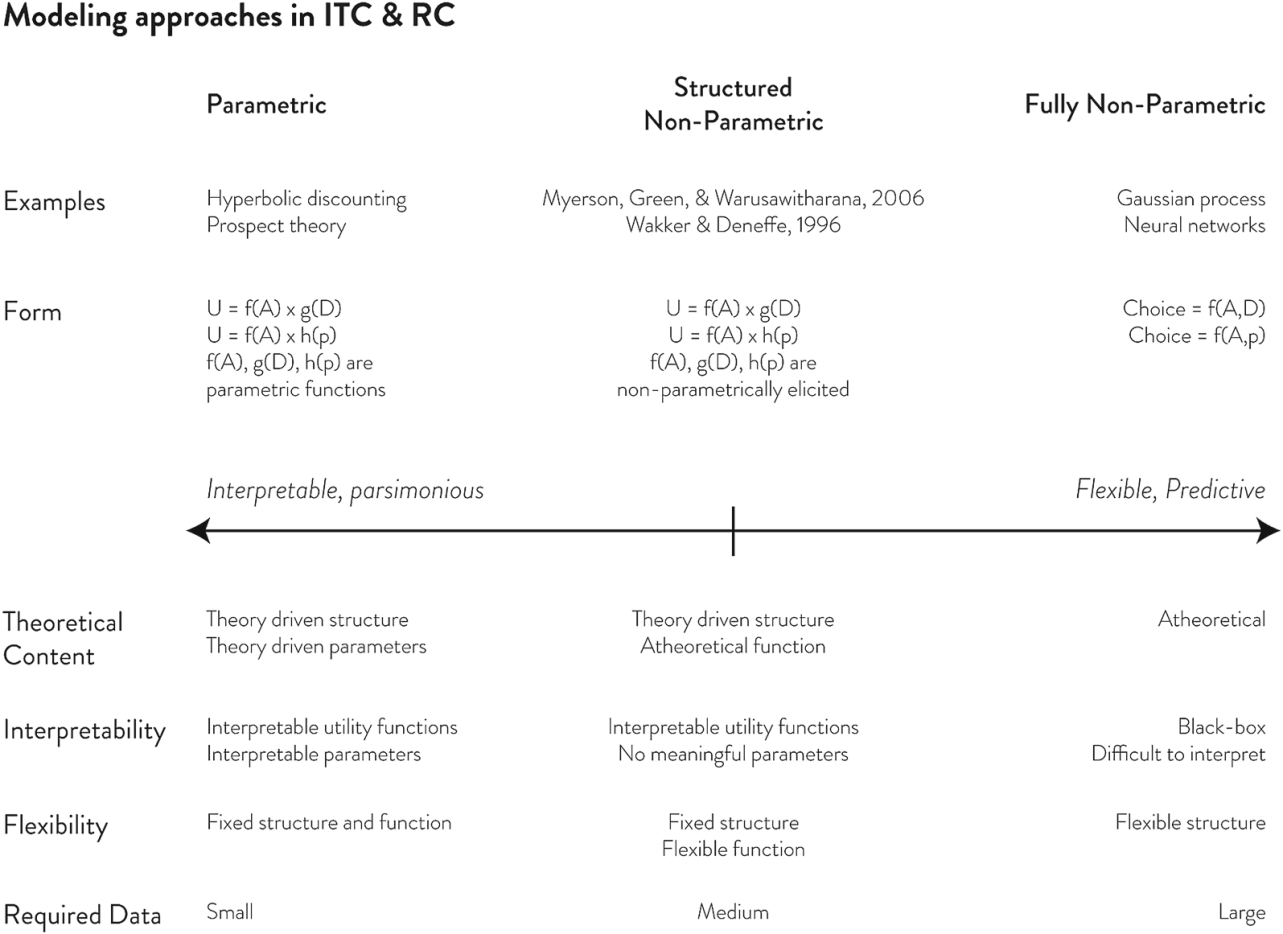
Three classes of modeling approaches in ITC & RC. Outlined above are characteristics of three different classes of modeling approaches to intertemporal choice and risky choice data. Parametric and fully non-parametric approaches have multiple tradeoffs in theoretical motivation, interpretability, flexibility, and required amount of data. Structured non-parametric approaches try to strike a balance between these two approaches

ITC and RC data are usually modeled using one of three ways: parametric, structured non-parametric, or fully non-parametric approaches (Fig. 1). The most popular approach uses parametric utility models to describe choice (e.g., Table 1). Its popularity is driven by two factors. First, parametric utility models can distill complex patterns of behavior into one or two interpretable parameters. For example, the discount rate parameter in ITC models represents the rate at which the value of future options declines with time delay (parameter *k* in Table 1); the risk-aversion parameter in RC models (often substituted by the value function curvature parameter: parameter $\alpha$ in Table 1) captures the deviation of utilities from risk-neutral expected value. These parameters are especially useful in applied research that seeks to correlate these measures with other variables such as health or intelligence. Obtaining these estimates, of course, requires fitting the model to data, which highlights a second benefit: minimal data requirements. Parametric models, owing to their simple forms, often do not require extensive choice datasets. They can be nested inside logit or probit choice models and fit to any dataset using simple procedures such as maximum likelihood estimation (MLE).

However, parametric models are not without drawbacks. Due to their simple form, parametric models have difficulty accounting for the heterogeneity in utility function shapes. Recent evidence shows that different people behave according to different utility models and that there is no ‘one correct model’ that can describe everyone’s behavior equally well (Bruhin et al. 2009; Cavagnaro et al. 2016; Franck et al. 2015; Myerson et al. 2006). Consequently, researchers must ascertain that their findings are not dependent on their choice of parametric model. To this end, they may have to perform the same analysis multiple times using different utility models to show the robustness of their results (e.g., Ballard and Knutson 2009; Kable and Glimcher 2007). However, not only is this an added burden, it is also an imperfect solution as there always could be another model to consider. In sum, while parametric models are useful in their simplicity and interpretability, their assumptions can be questionable at the individual level due to heterogeneous utility functions.

On the other side of the spectrum, there are fully non-parametric approaches (Fig. 1). With modern generalized prediction algorithms such as Gaussian processes, neural networks, etc., one can treat choice modeling as a classification problem without needing to specify any structure or functional form. Given that these algorithms were designed for the goal of prediction, it is expected that fully non-parametric approaches will be more predictive than parametric approaches. However, achieving this predictive power requires considerably more data. In a dataset of about 100 choices, Arfer and Luhmann (2015) found that support vector machines, random forests, and k-nearest neighbor clustering algorithms do *not* have higher predictive capabilities than parametric models. More importantly, these non-parametric methods are agnostic, ‘black-box’ approaches that do not readily yield interpretable insights, and therefore have rarely been used in studies seeking to advance theories of the decision-making processes involved in ITC and RC.

**Table 1:** Survey of commonly used ITC and RC models

Intertemporal choice models	Form name	Utility function	Approx. by CBS
Samuelson (1937)	Exponential (E)	$U=A\cdot \exp \left( -kD \right)$	Y
Mazur (1987)	Hyperbolic (H)	$U=A\cdot \left( 1+kD \right) ^{-1}$	Y
Green et al. (1994)	Generalized Hyperbolic (Gh)	$U=A\cdot \left( 1+kD \right) ^{-s}$	Y
Roelofsma (1996)	Log Time (Lt)	$U=A\cdot D^{-k}$	Y
Laibson (1997)	Quasi-hyperbolic (Q)	$U=A\cdot \beta \exp \left( -kD \right)$	Y
McClure et al. (2007)	Double Exponential (De)	$U=A\cdot \left( we^{-aD}+\left( 1-w \right) e^{-bD} \right)$	Y

Risky choice models	Form name	Utility function	Approx. by CBS
Von Neumann and Morgenstern (1945)	Expected Utility Theory (Eut)	$U=A^{\alpha }\cdot p$	*Y
Rachlin et al. (1991)	Hyperbolic (H)	$U=A\cdot \left( 1+h\left( \frac{1-p}{p} \right) \right) ^{-1}$	Y
Goldstein and Einhorn (1987)	GE-weight Prospect T. (Ge)	$U=A^{\alpha }\cdot \left( \frac{\delta p^{\gamma }}{\delta p^{\gamma }+\left( 1-p \right) ^{\gamma }} \right)$	*Y
Tversky and Kahneman (1992)	TK-weight Prospect T. (T)	$U=A^{\alpha }\cdot \left( \frac{p^{\gamma }}{\left( p^{\gamma }+\left( 1-p \right) ^{\gamma } \right) ^{\frac{1}{\gamma }}} \right)$	*Y
Prelec (1998)	Prelec-weight Prospect T. (P)	$U=A^{\alpha }\cdot \exp \left( -\delta \left( -\ln p \right) ^{\gamma } \right)$	*Y
Green and Myerson (2004)	Generalized Hyperbolic (Gh)	$U=A\cdot \left( 1+h\left( \frac{1-p}{p} \right) \right) ^{-s}$	Y
Markowitz (1959)	Risk-Return (R)	$U=A\cdot p-b\cdot Var$	N
Slovic and Lichtenstein (1968)	Attribute (A)	$U=\beta _{0}+\beta _{1}A+\beta _{2}p$	N
Weber et al. (2004)	Coefficient of Variation (C)	$U= A\cdot p-b\cdot CV$	N

Each row shows, from left to right, the reference of the parametric model, the name of the form (with short abbreviation), the model specification, and whether the model can be approximated by a CBS function of the form in this paper. Across all ITC models, utility is expressed as a product of A, the amount of the delayed outcome, and f(D), which is a function of the delay (we are assuming a linear utility for amount in ITC; to the extent to which this assumption is violated, the functions we estimate will incorporate influences of both amount and delay transformations, much like some of the RC models). In RC models, A is the amount of the risky outcome, p is the probability of winning that outcome. We only show here the model forms for a simple gamble in which there is a probability p of winning A and probability 1-p of winning nothing. The RC models marked with an asterisk are approximated by CBS in their analytically converted form of $U= A\cdot f(p)$ (see supplemental materials section A for the conversion proof and see Table 2 for the converted form).

Balancing the interpretability of parametric approaches and the flexibility of non-parametric approaches are structured non-parametric approaches (Fig. 1). Structured non-parametric approaches keep the same overall structure of the parametric utility functions (e.g., $U=f\left( A \right) *g\left( D \right)$, where ITC utility is modeled as a product of transformed amount and delay, or $U=f\left( A \right) *h\left( p \right)$, where RC utility is modeled as a product of transformed amount and probability), but approximate these transformation functions in a non-parametric manner. Hence, compared to parametric approaches, there is greater flexibility, while compared to fully non-parametric approaches, there is greater interpretability since these transformation functions are understood as weighting functions for amount, delay or probability. Furthermore, previous research has shown that the area under the curve (AUC) of these non-parametrically fitted functions can serve as measures of impulsivity or risk-aversion in lieu of the simple scalar discount rate or risk-aversion parameters from parametric models (Myerson et al. 2006).

Unfortunately, current structured non-parametric approaches have an important drawback that limits their widespread use: they require specialized elicitation procedures. In ITC, an adaptive experimental design has been used to directly estimate the discounting function *g*(*D*) at a few given delays (Myerson et al. 2006). Hence, this approach cannot be used post-hoc on choice datasets that do not have the same structure. In RC, specialized elicitation procedures have been designed to address the problem that the commonly used prospect theory form of $U=f\left( A \right) *h\left( p \right)$ is not identifiable in most choice datasets even for parametric functions. For example, using a power value function for amount, $f\left( A \right) =A^{\alpha }$ , and Prelec’s (1998) 2-parameter probability weighting function, $h\left( p \right) =e^{-\delta \left( -\ln p \right) ^{\gamma }}$, a certain smaller monetary option (SA) is equivalent in utility to a larger risky monetary option (LA) with probability *p* when $SA^{\alpha }=LA^{\alpha }\cdot e^{-\delta \left( -\ln p \right) ^{\gamma }}$. Note that all terms in this equivalence relationship have exponents that can be arbitrarily increased or decreased while maintaining the equality (e.g., $SA^{2\alpha }=LA^{2\alpha }\cdot e^{-2\delta \left( -\ln p \right) ^{\gamma }})$, showing that the value function parameter $\alpha$ and the weighting function elevation parameter $\delta$ can tradeoff in their effects on choice. To get around this problem with identifiability, Wakker and Deneffe (1996) and Abdellaoui (2003) carefully constructed choice sets to mathematically cancel out the effect of $f\left( A \right)$ or $h\left( p \right)$ so that the other function can be estimated without being confounded. This ingenious method, however, requires a specifically constructed choice set that is quite cognitively demanding. Thus, in both ITC and RC, existing structured non-parametric approaches require specialized elicitation procedures that can limit their widespread use.

Here we provide a novel structured non-parametric approach that can be used on any dataset. We use a model-based approach that uses cubic Bezier splines (CBS; de Casteljau, 1963) to approximate smooth monotonic variable transformation functions that can be fitted with MLE. We also provide the statistical package in MATLAB and R to be used for future research. The MATLAB package is available on github (https://github.com/sangillee/CBSm), and the R package can be downloaded from CRAN under the package name ‘CBSr’ (https://CRAN.R-project.org/package=CBSr) to allow researchers to reproduce and extend our results.

In this paper, consistent with the role for structured non-parametric approaches outlined in Fig. 1, we demonstrate both the predictive advantages (compared to parametric approaches) and interpretive advantages (compared to fully non-parametric approaches) of CBS. Predictive performance is assessed in two ways. First, we show via simulation that CBS does not require substantially larger amounts of data compared to parametric methods. Second, using an empirical dataset of ITC and RC, we show that CBS has higher in-sample and out-of-sample predictive power compared to various parametric methods. The interpretive benefits of CBS are also demonstrated in two ways. First, we show that CBS can yield interpretable insights into exactly why it has higher predictive power compared to parametric methods, thereby pointing to new paths for theoretical models to be developed. Second, we show that CBS can yield reliable estimates of individual impulsivity and risk-aversion that are consistent across time, thereby providing an alternative method to measure these individual traits without using parametric models.

**Fig. 2:**
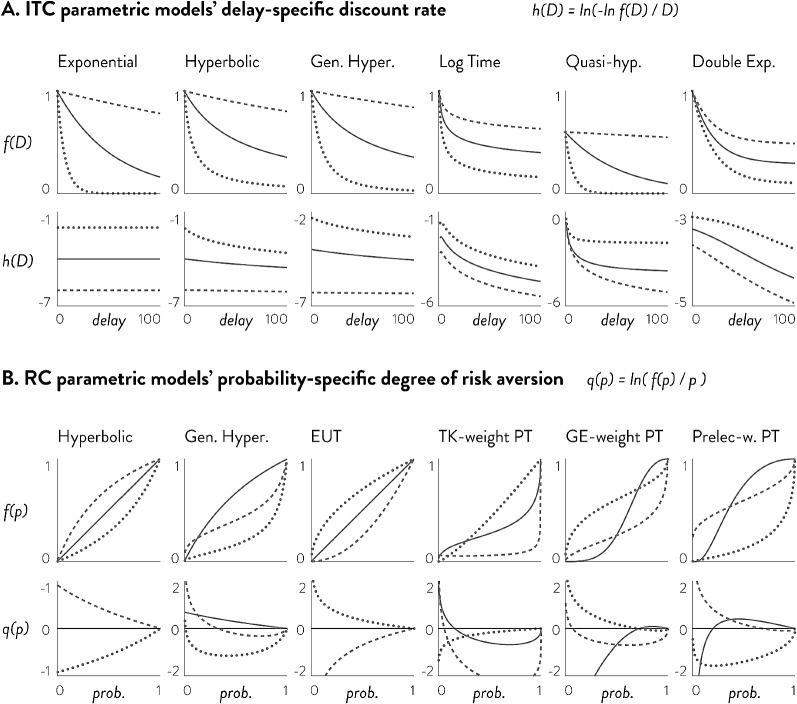
Delay-specific discount rates and probability-specific degrees of risk aversion for different parametric models. **a** is the delay-specific discount rate of ITC models in Table 1. All parametric models of ITC in consideration show either constant (exponential) or decreasing delay-specific discount rates. **b** is the probability-specific degree of risk aversion, which is the log of the ratio between objective and subjective probabilities. A measure above 0 would indicate over-appreciation of probabilities and hence risk-seeking, while a measure below 0 would indicate risk-aversion. All parametric models of RC in consideration assume a behavioral pattern that switches between risk-aversion and risk-seeking at most once. In other words, the probability-specific degree of risk aversion for all RC parametric models can cross 0 (risk-neutral point) at most once

Specifically, the higher predictive power of CBS comes from capturing patterns of discounting and risk aversion that violate the assumptions of most existing parametric models. Existing parametric models of ITC typically assume constant or decreasing discount rates over time. The discount rate at a given delay $D^{*}$ can be calculated as $h( D^{*} )=\ln (-\ln (f\left( D^{*} \right) )/D^{*})$, which is a constant in the case of the exponential function: ln(–ln($\hbox {e}^{-kD^{*}})/D^{*})= \ln (k)$. All other common models, as shown in Fig. 2a, show decreasing discount rates over time. Existing parametric models of RC typically assume that people alternate between risk-averse and risk-seeking behavior no more than once across probabilities. If we convert RC models into a discounting form of $U=A\cdot f\left( p \right)$, we can measure the degree of risk-aversion at a given probability $p^{*}$ by $q\left( p^{*} \right) =\ln \left( f(p^{*})/p^{*} \right)$, which is the log odds of subjective to objective probabilities. As shown in Fig. 2b, expected utility theory and hyperbolic models assume that people are risk-averse or risk-seeking throughout all probabilities, while prospect theory models and generalized hyperbolic models assume that people’s behavior can ‘switch’ at most once from risk-seeking to risk-aversion (or vice versa) as probabilities increase (indicated by the change of sign in $q\left( p^{*} \right) )$. We show that CBS’s main predictive benefits are derived from participants who show increasing discount rates over time in ITC and who switch multiple times between risk-aversion and risk-seeking across probabilities in RC.

## Cubic Bezier Splines Model Specification

We consider structured non-parametric estimation of the form $U=A\cdot f\left( X \right)$; in ITC, this would be $U=A\cdot f\left( D \right)$ where amount (A) is discounted as a function of delay (D), and in RC, this would be $U=A\cdot f\left( p \right)$ where amount (A) is discounted as a function of probability (*p*). The discounting form has several benefits. First, most ITC models are already in discounting form, which allows our approach to approximate them well. Second, even for models where the amount is also transformed (i.e., $U=f\left( A \right) \cdot g\left( X \right) )$, one can analytically convert them into the discounting form. This includes some ITC models that have amount transformations and many RC models such as prospect theory. Hence in this case, our discounting function would measure the combined effect of both transformation functions (see supplemental materials A for details on model conversion). Third, the discounting form is easily identifiable through choice data, unlike prospect theory forms, which, as previously mentioned, are hard to identify. Finally, the discounting form allows a measure of impulsivity and risk aversion to be solely contained in one fitted function, which makes interpretation of the utility function easy. It is important to note, however, that this form cannot capture all classes of parametric models; for example, it cannot approximate mean-variance-type models of RC or attribute comparison-type models (Table 1). Nevertheless, the discounting form covers a large number of extant parametric models and allows for easy estimation of impulsivity and risk aversion via AUC. Embedding this discounted utility function inside a binary logit choice model gives us the following specification:

1 $$\begin{aligned} \log \left( \frac{p\left( \hbox {choice}_{t}=1 \right) }{p\left( \hbox {choice}_{t}=2 \right) } \right) =\sigma \left( U_{1t}-U_{2t} \right) ,\quad U_{jt}=A_{jt}\cdot f\left( X_{jt} \right) ,\quad j=1,2 \end{aligned}$$

where $$\sigma $$ is a free parameter that determines the relationship between the scale of the utilities ($$U_{1t}$$, $$U_{2t})$$ and choice, and $$X_{jt}$$ is either delay or probability, depending on the task. The subscript *j* denotes the two options (1 and 2), and the subscript *t* denotes the trial number. Hence, the key question comes down to this: how to flexibly approximate $$f\left( X_{jt} \right) $$?

In approximating $f\left( X_{jt} \right)$, we seek to incorporate two normative constraints: smoothness and monotonicity. Given a continuously smooth input variable such as delay or probability, it makes normative sense that the output variable of utility is also continuously smooth. In ITC, it makes normative sense for utility to decline monotonically as a function of delay, while in RC, to increase monotonically as a function of probability. The two normative constraints of smoothness and monotonicity are already implicit in almost all of the existing parametric utility models and can serve as important priors that combat over-flexibility. Hence, the goal was to estimate a smooth, monotonic univariate transformation of $f\left( X_{jt} \right)$. However, the monotonicity constraint makes the use of several methods difficult. Polynomial or Fourier basis regressions, while continuously smooth, control the flexibility of the curve by changing the order of the equation, which unfortunately also changes the order of the derivative and complicates the constraining problem (see supplemental materials B for discussion on B-splines). Hence, we find instead that by chaining multiple pieces of cubic-order Bezier splines, each of them separately monotonically constrained, we can approximate *f*(*X*) in a smooth, monotonic manner, without requiring specialized datasets.

**Fig. 3:**
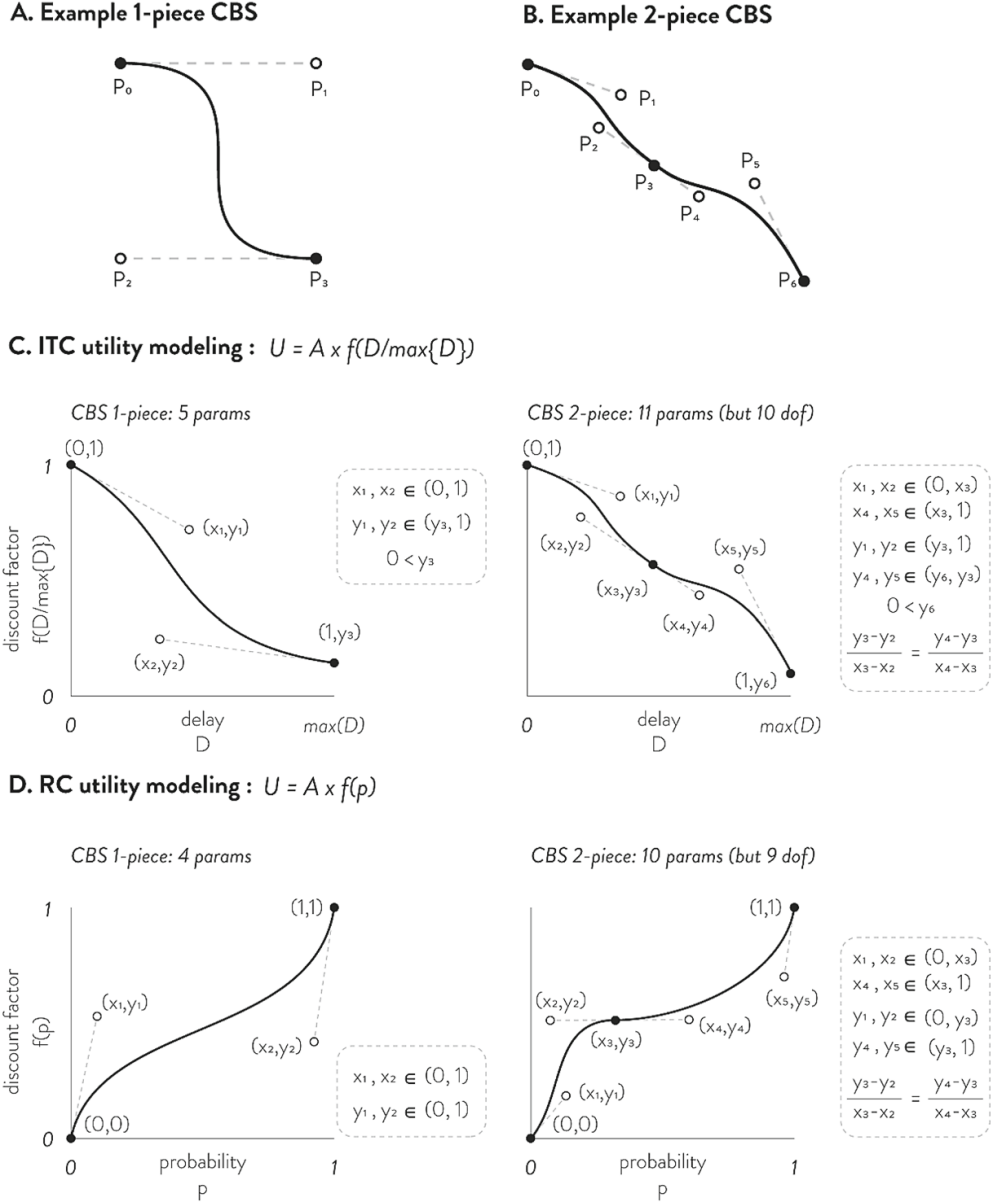
Example 1-piece and 2-piece CBS (**a** and **b**, respectively), and model specification of ITC (**c**) and RC (**d**) using 1-piece (left) and 2-piece CBS (right). Example 1-piece CBS is shown in (**a**), and 2-piece CBS is shown in (**b**). While each piece requires 4 points, because adjoining points overlap, 2-piece CBS requires 7 points. **c** Shows how CBS is used to flexibly model the delay discounting function and **d** shows how CBS is used to flexibly model the probability weighting function. In both ITC and RC, the coordinates of the points are free parameters that are estimated. The parameter constraints are shown on the right of each panel in dotted boxes. In the case of 2-piece CBS, there is one less degree of freedom than number of parameters due to the necessity of ($$x_{{2}},y_{{2}})$$, ($$x_{{3}},y_{{3}})$$, and ($$x_{{4}},y_{{4}})$$ being on the same line

Piecewise-connected CBS are already widely used in graphics software, fonts, and interpolations, but have seen limited use as function approximators compared to other types of splines. This is because while most splines are defined in the form of $$y = f(x)$$, where the *y*coordinate is expressed as a function of *x*, CBS’s functional form is much more general: both the *x* and *y* coordinates are independently expressed as functions of a third variable *t*. A single piece of CBS is defined by four points ($$P_{0x}$$, $$P_{0y})$$, ($$P_{1x}$$, $$P_{1y})$$, ($$P_{2x}$$, $$P_{2y})$$, ($$P_{3x}$$, $$P_{3y})$$ (Fig. 3a). The coordinates of these four points become the parameters of the CBS as the *x* and *y*-coordinates of the spline are controlled independently by two separate cubic functions.

2 $$\begin{aligned} x= & {} m\left( t \right) = \left( 1-t \right) ^{3}P_{0x}+3\left( 1-t \right) ^{2}tP_{1x}+3\left( 1-t \right) t^{2}P_{2x}+t^{3}P_{3x}, \quad 0\leqslant t\leqslant 1 \end{aligned}$$

3 $$\begin{aligned} y= & {} n\left( t \right) = \left( 1-t \right) ^{3}P_{0y}+3\left( 1-t \right) ^{2}tP_{1y}+3\left( 1-t \right) t^{2}P_{2y}+t^{3}P_{3y}, \quad 0\leqslant t\leqslant 1 \end{aligned}$$

These two functions can jointly be used to approximate the function $f\left( D \right)$ in ITC or $f\left( p \right)$ in RC by $y=n\left( m^{-1}\left( x \right) \right)$ as long as $x=m(t)$ and $y=n\left( t \right)$ are both monotonic functions of *t*. We find that the constraint for monotonicity is very simple: if the *x* and *y* coordinates of the two middle points (P1 and P2) stay between that of the end points (P0 and P3), the resulting CBS is monotonic (i.e., $P_{1x},P_{2x}\in \left[ P_{0x},P_{3x} \right]$, and $P_{1y},P_{2y}\in \left[ P_{0y},P_{3y} \right]$; see supplemental materials C, D, E for proof). It is also important to note that the CBS’s local derivative at the end point equals the slope of the line connecting the end point with its neighboring point (i.e., $\bar{P_{2}P_{3}}$ in Fig. 3a). Using this property, multiple pieces of CBS can be smoothly joined by equating the local derivative (i.e., ensuring that three points $P_{2} P_{3} P_{4}$ are on the same line in Fig. 3b). Figure 3c, d shows the CBS parameters involved in modeling *f*(*D*) and $f\left( p \right)$ in ITC and RC using either 1-piece or 2-pieces of CBS.

Because the CBS form ($y=n\left( m^{-1}\left( x \right) \right)$, Eqs. 2 and 3) cannot be succinctly expressed as $y=f(x)$, the likelihood function for the choice model using CBS also cannot be succinctly expressed. Instead, shown below are the general MLE steps (a pseudo-algorithm) used to fit a CBS-based choice model:

1. Start with some initial CBS points (Fig. 3 shows relevant points for each case)
2. For all $X_{jt}$ (delay or probability), find $t_{jt}^{*}$ that satisfies $X_{jt}=m\left( t_{jt}^{*} \right)$ as given in Eq. 2. In our statistical package, we use a numerical search since the root is bounded within [0 1] and the analytical roots are unstable and computationally costly due to radicals and transcendentals (depending on which cubic formula is used).
3. Then, calculate $U_{jt}=A_{jt}\cdot n(t_{jt}^{*})$ as given in Eq. 3
4. Use Eq. 1 to calculate the log-likelihood of all choices
5. Propose new parameters using gradient descent while maintaining constraints in Fig. 3. This can be done using a general-purpose optimization tool that supports linear and nonlinear constraints using Lagrangian multipliers. In this paper, we used MATLAB’s optimization tool (fmincon).
6. Repeat step 2 through 5 until convergence

For this paper, we only entertain 1piece and 2piece CBS as they seem sufficient in approximating the parametric utility models shown in Table 1. All empirical and simulated data as well as analysis codes are included in this article in its supplementary information files.

**Table 2:** Simulating utility functions for CBS recovery

Simulating function	Equivalent expression in $U=A\cdot f(X)$ form	Simulating parameters
Exponential	$f\left( D \right) =\exp \left( -kD \right)$	$\ln k\in \left\{ -8,-6,-4,-2 \right\}$
Hyperbola	$f\left( D \right) =\left( 1+kD \right) ^{-1}$	$\ln k\in \left\{ -8,-6,-4,-2 \right\}$
General Hyp.	$f\left( D \right) =\left( 1+kD \right) ^{-s}$	$\left( \ln k,s \right) \in \left\{ \left( -7,0.5 \right) ,\left( -7,2 \right) ,\left( -4,0.5 \right) ,\left( -4,2 \right) \right\}$
Logarithmic Time	$f\left( D \right) =D^{-k}$	$k\in \left\{ 0.4,0.2,0.1,0.05 \right\}$
Quasi-hyperbolic	$f\left( D \right) =\beta \exp \left( -kD \right)$	$\left( \beta ,\ln k \right) \in \left\{ \left( 0.4,-7 \right) ,\left( 0.4,-4 \right) ,\left( 0.8,-7 \right) ,\left( 0.8,-4 \right) \right\}$
Double Exp.	$f\left( D \right) =we^{-aD}+\left( 1-w \right) e^{-bD}$	$\ln a=-8,\ln b=-3,w\in \left\{ 0.7,0.5,0.3,0.1 \right\}$
EUT	$f\left( p \right) =p^{1/\alpha }$	$\alpha \in \left\{ 0.2,0.6,1,2 \right\}$
Hyperbola	$f\left( p \right) =\left( 1+h\left( p^{-1}-1 \right) \right) ^{-1}$	$h\in \left\{ 0.1,0.5,2,7 \right\}$
GE weighting	$f\left( p \right) =\left( \frac{\delta p^{\gamma }}{\delta p^{\gamma }+\left( 1-p \right) ^{\gamma }} \right) ^{1/\alpha }$	$\alpha =0.8,\left( \delta ,\gamma \right) \in \left\{ \left( 0.5,0.5 \right) ,\left( 0.5,2 \right) ,\left( 2,0.5 \right) ,\left( 2,2 \right) \right\}$
TK weighting	$f\left( p \right) =\left( \frac{p^{\gamma }}{\left( p^{\gamma }+\left( 1-p \right) ^{\gamma } \right) ^{\frac{1}{\gamma }}} \right) ^{1/\alpha }$	$\alpha =0.8,\gamma \in \left\{ 0.25,0.5,1,3 \right\}$
Prelec weighting	$f\left( p \right) =\exp \left( -\frac{\delta }{\alpha }\left( -\ln p \right) ^{\gamma } \right)$	$\alpha =0.8,\left( \delta ,\gamma \right) \in \left\{ \left( 0.5,0.5 \right) ,\left( 0.5,2 \right) ,\left( 2,0.5 \right) ,\left( 2,2 \right) \right\}$
General Hyp.	$f\left( p \right) =\left( 1+h\left( p^{-1}-1 \right) \right) ^{-s}$	$\left( h,s \right) \in \left\{ \left( 2,0.3 \right) ,\left( 7,0.3 \right) ,\left( 2,1.5 \right) ,\left( 7,1.5 \right) \right\}$

Shown above are the ITC models and RC models used for assessing CBS’ function recovery expressed in $U=A\cdot f\left( X \right)$ form (see Supplemental Materials A for transformation proof). The parameter sets used to simulate choice datasets are shown on the right column.

## Methods

### Predictive Accuracy

We assess the predictive capacity of CBS in two ways. First, we simulate choice data from various parametric utility functions to examine how well CBS can recover the true functions at different dataset sizes. We simulate binary choices from 6 models for ITC and 6 models for RC, each with 4 parameter combinations. The chosen models and their 4 parameter combinations are shown in Table 2. Each simulated choice is between a smaller monetary amount of $20 (fixed across all trials), and a larger monetary amount that varies from trial to trial. The larger monetary amount is either delayed (for ITC models) or probabilistic (for RC models). The amount of the larger monetary option on each trial is created by uniformly sampling the ratio between the smaller and larger monetary amount ($$0 \sim 1$$; e.g., ratio of 0.5 means the smaller amount is half that of the larger amount). In ITC simulations, the delays are uniformly sampled from $$0\sim 180$$ days, and in RC simulations, the probabilities are uniformly sampled from 0 to 1. Dataset sizes range from $$7^{{2}}= 49$$ to $$20^{{2 }}= 400$$ choices based on how finely we sample the range of delay/probability and amount. The difference in utilities of the two options is used in a logit model to generate choice probabilities, according to which we generate binary choices:

4 $$\begin{aligned} \log \left( \frac{p\left( \hbox {choice}_{t}=1 \right) }{p\left( \hbox {choice}_{t}=2 \right) } \right) =\sigma \left( U_{1t}-U_{2t} \right) \end{aligned}$$

where $$\sigma $$ models the overall scale of the utility difference between the two options. For simulation, the scaling parameter $$\sigma $$ is fixed at 1, as it is not a variable of interest in our study. The utilities of each option on each trial ($$U_{1t}, U_{2t})$$ are modeled according to the forms shown in Table 2. For each of the $$(6+6) \times 4 = 48$$ functions x 14 dataset size conditions $$(7^{{2}}\sim 20^{{2}})$$, we simulate 200 datasets. All simulated datasets are then fitted with the 6 parametric models and CBS (both 1-piece and 2-piece). We measure the mean absolute error (MAE) between the fitted functions and the true simulating functions to assess each model’s recovery of the true functions. Since the error is measured relative to the true function, the MAE here is best interpreted as an out-of-sample measure; it is not given that more flexible models will have lower MAEs as it may overfit the choice noise instead of the true function. Rather, simple models may have lower MAEs in smaller datasets, while more complex models may have lower MAEs in larger datasets due to the bias-variance tradeoff. The key question is at which dataset size (if any) CBS, the more complex model, outperforms parametric models, which are simpler.

Our second assessment of predictive capacity comes from in-sample and out-of-sample prediction in real ITC and RC data. We utilize ITC and RC data collected in Kable et al. (2017). 166 participants completed binary choice tasks in ITC and RC and 128 of them returned after 10 weeks to perform the same task again in session 2. In each session, participants made 120 binary choices each in the ITC task and RC task. The choices in the ITC tasks were between a smaller immediate monetary reward that was always $20 today (i.e., the day of the experiment) and a larger later monetary reward (e.g., $Y in D days; D $$\sim $$ [20 180], Y $$\sim $$ [22,85]). The choices in the RC tasks were between a smaller certain monetary reward that was always $20 and a larger probabilistic monetary reward (e.g., $Y with probability *p*;$$ p \sim $$ [.09 .98], Y $$\sim $$ [21 85]). We treat session 1 and 2 as if they are separate participants and only include sessions with at least two or more of each choice type (i.e., at least two smaller reward choices and two larger reward choices in 120 trials), which rules out 9 sessions for ITC and 4 sessions for RC. This is because at least two of each choice type is necessary for leave-one-trial-out cross-validation; otherwise the training dataset may have entirely one-sided choices (i.e., all smaller reward choices or all larger reward choices).

In the empirical data, we compare the descriptive and predictive capabilities of CBS against other parametric models in Table 1. For all models (including CBS), we measure their in-sample and out-of-sample prediction accuracies and Tjur’s D. Tjur’s D (coefficient of discrimination) is the difference of the mean choice probabilities of each choice type. For example, a good model of ITC should have high *p*(*delayed choice*) for delayed choices but low *p*(*delayed choice*) for immediate choices. Hence, the difference between the mean of those two choice probabilities is bounded between 0 (random model) and 1 (perfect model) and tells how well the two choice types are discriminated in out-of-sample predictions. Even if two models have the same hit rate accuracy, Tjur’s D is higher for models that classify the trials with larger discrimination in choice probabilities. All models are fitted at the individual level, and out-of-sample prediction is performed using a leave-one-out cross-validation (LOOCV) procedure where the model is fit on all but one trial of the data and used to predict the left out trial. This cross-validation procedure allows us to maximally retain the training dataset size for each individual, since k-fold or hold-out cross-validation would require substantially reducing the size of the training dataset. All models are fit using a logit choice model (Eq. 4). We have also tried a linear probability model (LPM) specification for choice and, to the extent we have evaluated it, all our main conclusions also hold under a LPM. These comparative results are available from the authors upon request.

### Interpretability

We demonstrate two ways in which CBS fits yield interpretable insights. First, we use CBS to demonstrate novel patterns of behavior that are not captured by extant parametric models. We achieve this by quantitatively calculating the delay-specific discount rates and probability-specific risk-aversion of each individual and assessing the patterns in which CBS has higher predictive performance over extant parametric methods. Using the out-of-sample prediction values of Tjur’s D, we show that a portion of participants are better fit by CBS models because they exhibit behavior violating the assumptions of extant parametric models. Specifically, we show that, in ITC, some participants exhibit increasing discount rates over time, and in RC, some participants exhibit multiple alternations between risk aversion and risk seeking across probabilities.

Second, we use CBS to obtain measures of impulsivity and risk-aversion without assuming a parametric utility model. The CBS measures of impulsivity and risk-aversion can be obtained by measuring the area under the curve (AUC) of the fitted CBS function (see supplemental materials F for analytic expression). Since CBS models ITC and RC utility in discounting form ($U=A\cdot f\left( X \right) )$, the AUC of the discounting function $f\left( X \right)$ serves as a measure of how much the amount is discounted as a function of delay or probability. In previous research, AUC of discounting form utility functions has been proposed and used as a measure of impulsivity and risk-aversion in non-parametric utility estimation (Myerson et al. 2006). We show that the AUC of CBS fits can serve as stable, subject-specific measure of impulsivity and risk-aversion, just like the parameter estimates of extant parametric models, by testing cross-session consistency (i.e., correlation) of ITC and RC AUC. We also provide the standard error of the AUC estimates by performing a jackknife resampling procedure.

**Fig. 4:**
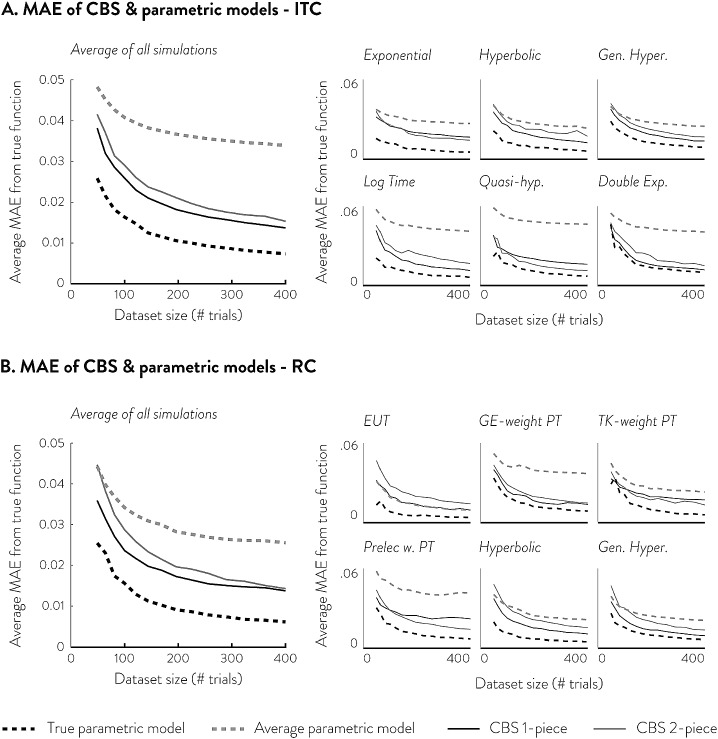
Choice dataset simulations and recovery results. **a**, **b** Shows the average MAE of parametric and CBS functions under different simulating utility functions for ITC and RC, respectively. The large graphs on the left side show the average MAEs across all six simulating functions, while the small graphs on the right side show them for each of the six simulating functions separately. The dotted line shows the MAE of parametric models, while the solid line shows the MAE of CBS models. The dark dotted line shows the MAE of correctly specified parametric models, which serves as the theoretical lower bound of MAE at different dataset sizes

## Results

### Utility Function Recovery & Dataset Size

CBS shows excellent recovery of various latent utility functions even at small dataset sizes. Figure 4 shows the mean absolute error (MAE) of CBS and parametric fits to various simulating utility functions at various dataset sizes. The MAEs of correctly specified parametric models (shown in dark dotted lines) serve as the empirical lower bound of MAE but are unlikely to be achieved in real data since we cannot know the true generating function. The average MAEs of parametric models (shown in grey dotted lines) serve as the estimated error one would expect to get by using any one of the parametric utility models in Table 2 when the underlying choice data has heterogeneity and is generated from various utility functions. Due to formal similarities between many parametric models, the average MAEs of parametric models are not too big, nor do they vary greatly. Nevertheless, in both ITC and RC, the estimation error of CBS functions is lower than the average estimation errors of parametric models, even for smaller dataset sizes of 49 choices. This suggests that even in small dataset sizes, the estimation error one would get from using a parametric model (that is correct 1/6th of the time) is greater than the estimation error one would get from using CBS. We may, however, see that the error of 2-piece CBS functions is greater than parametric models in even smaller datasets, especially in RC. Also, generally we find that 1-piece CBS functions have lower estimation errors than 2-piece CBS functions except for certain simulating functions where 1-piece does not provide sufficient flexibility (see supplemental materials G for average CBS fits compared against true functions that can illuminate which functions required 2-piece CBS fits).

**Fig. 5:**
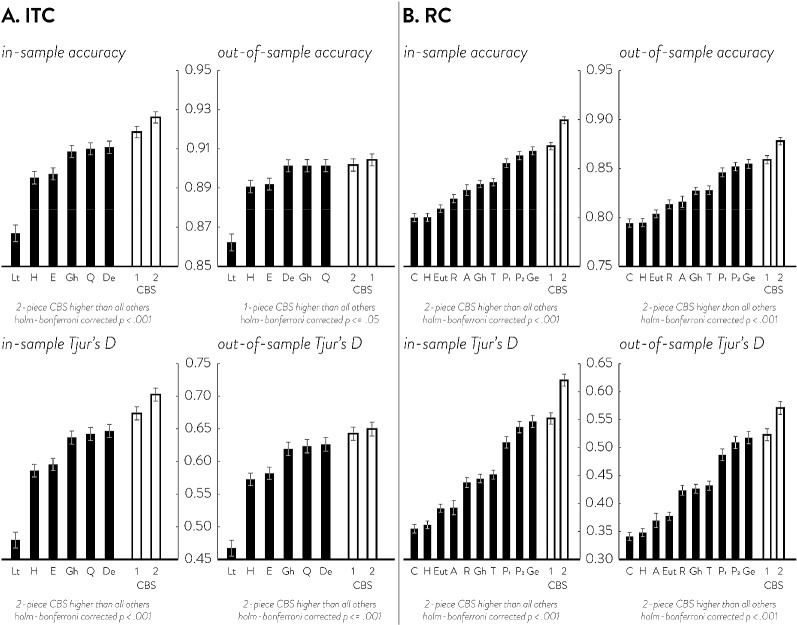
In-sample and out-of-sample prediction performance in ITC (**a**, left), and RC (**b**, right). In ITC, 6 parametric models and 2 CBS models are assessed; in RC, 10 parametric models and 2 CBS models are assessed. In both in-sample and out-of-sample, each model’s accuracy (top row) and Tjur’s D (bottom row) are assessed. The error bars represent the standard error of the mean

### Increased Descriptive & Predictive Power

In an empirical dataset of 120 choices, CBS shows higher in-sample and out-of-sample accuracies and Tjur’s D than all of the tested parametric models (Fig. 5). For both in-sample accuracy and Tjur’s D, we find that the 2-piece CBS function provides performance superior to all other methods in both ITC and RC, followed by 1-piece CBS. These in-sample results are somewhat expected given that models with more parameters are generally more likely to provide higher performance metrics. However, even in out-of-sample prediction CBS provides the highest accuracy and Tjur’s D compared to all other parametric models in both ITC and RC. This clearly demonstrates that CBS is not simply providing a flexible function that overfits empirical data; rather its flexibility is important in capturing individual characteristics so as to increase descriptive and predictive power. In ITC, out-of-sample accuracy is highest for the 1-piece CBS model followed by the 2-piece CBS model, while out-of-sample Tjur’s D is highest for the 2-piece CBS model followed by the 1-piece CBS model. This may suggest that while the 1-piece CBS model may provide the highest hit rate accuracy, the 2-piece CBS model may be able to better separate the two choice types. In RC, both out-of-sample accuracy and Tjur’s D are highest for the 2-piece CBS model, by a substantial margin over the next runner-up 1-piece CBS model. This pattern may suggest that RC data may generally require more complex functions than ITC data in order to adequately model behavior.

**Fig. 6:**
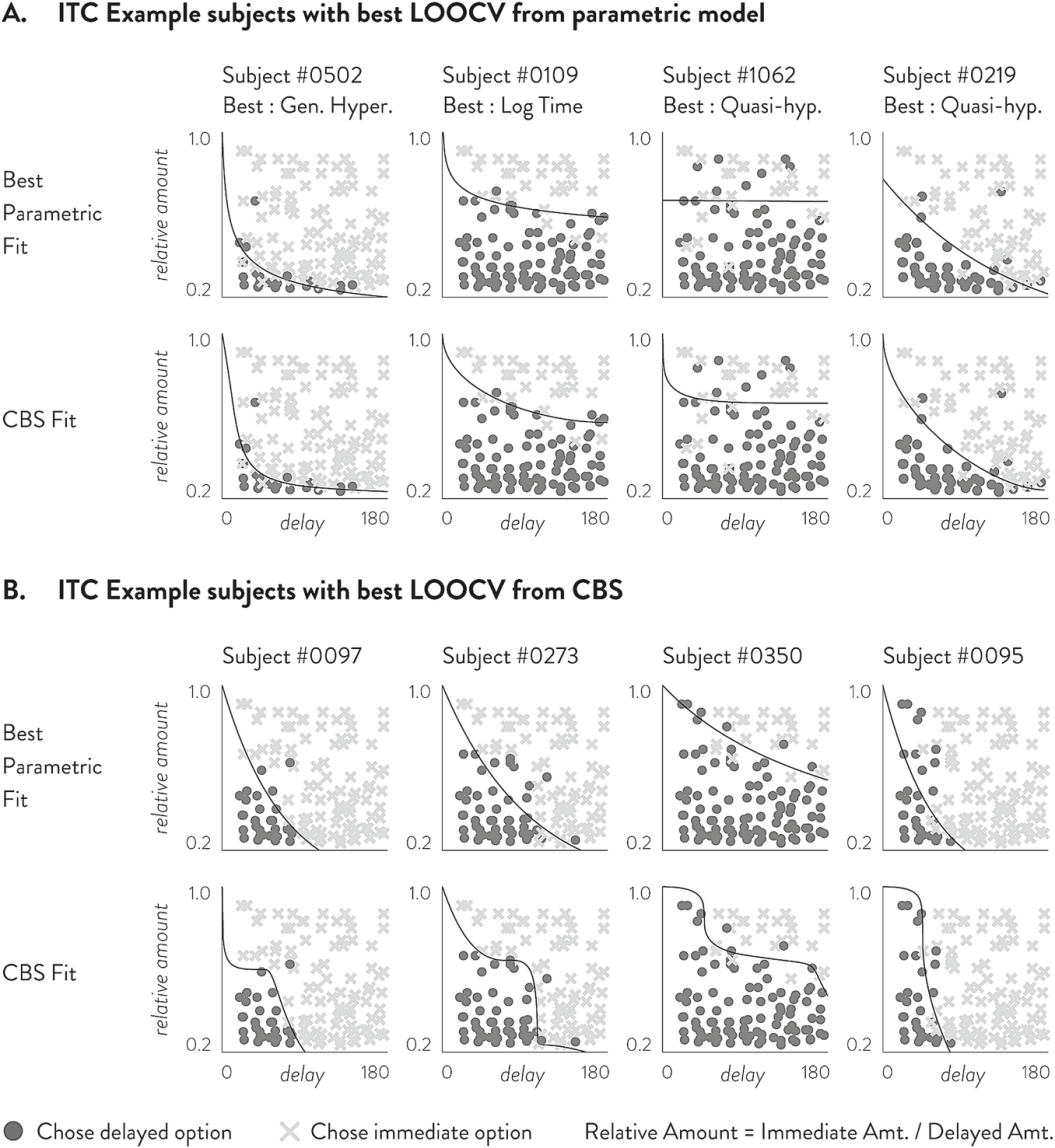
Plots of eight example participants’ choices in ITC, their best parametric fits and their best CBS fits as determined by LOOCV. **a** Shows 4 participants whose highest LOOCV Tjur’s D came from parametric models and **b** shows 4 participants whose highest LOOCV Tjur’s D came from CBS. In each panel, the top row shows the best parametric model (by LOOCV) and the bottom row shows the CBS fit. **a** participants are selected such that the diverse parametric forms can be shown; **b** participants are selected to show a variety of CBS fits that did not conform to parametric forms

**Fig. 7:**
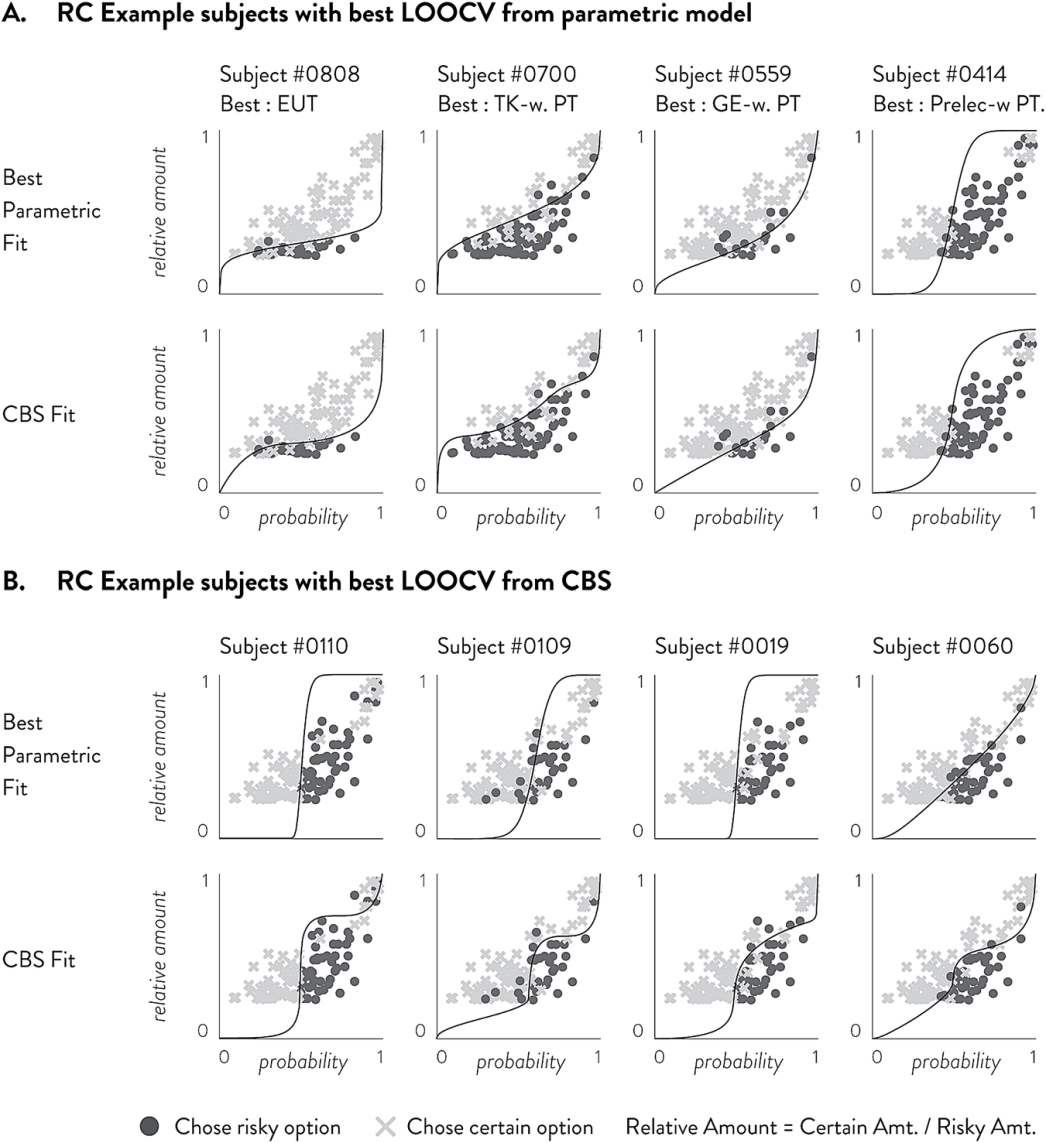
Plots of eight example participants’ choices in RC, their best parametric fits and their best CBS fits as determined by LOOCV. **a** Shows 4 participants whose highest LOOCV Tjur’s D came from parametric models and **b** shows 4 participants whose highest LOOCV Tjur’s D came from CBS. In each panel, the top row shows the best parametric model (by LOOCV) and the bottom row shows the CBS fit. **a** Participants are selected such that the diverse parametric forms can be shown; **b** Participants are selected to show a variety of CBS fits that did not conform to parametric forms

**Fig. 8:**
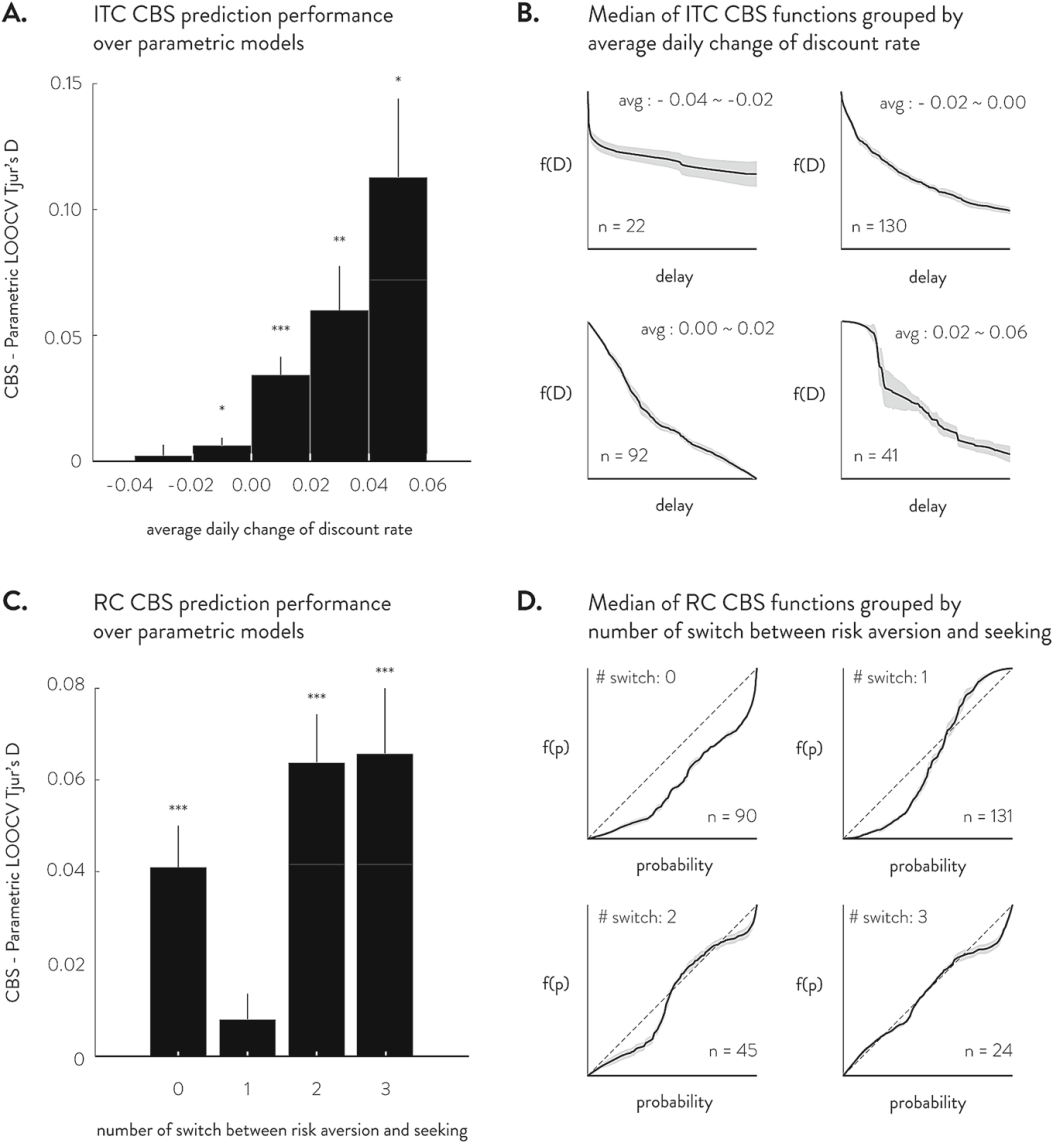
Deviation from common parametric forms. **a** Shows CBS prediction performance minus the maximum of parametric models’ prediction performance in ITC. CBS shows increasingly better predictions as the average daily change in discount rate becomes positive. **b** Shows the average fitted CBS functions for ITC grouped by the average daily change in discount rate. The solid line is the median function, with gray shade showing the standard errors. **c** Shows that, in RC, CBS provides better predictions in participants who do not alternate between risk aversion and risk seeking or alternate more than one time. Panel D shows the average fitted CBS functions for RC grouped by the number of switches between risk-aversion and risk-seeking behavior. *t test against 0, $p< .05$. **$p< .01$, ***$p <.001$

**Fig. 9:**
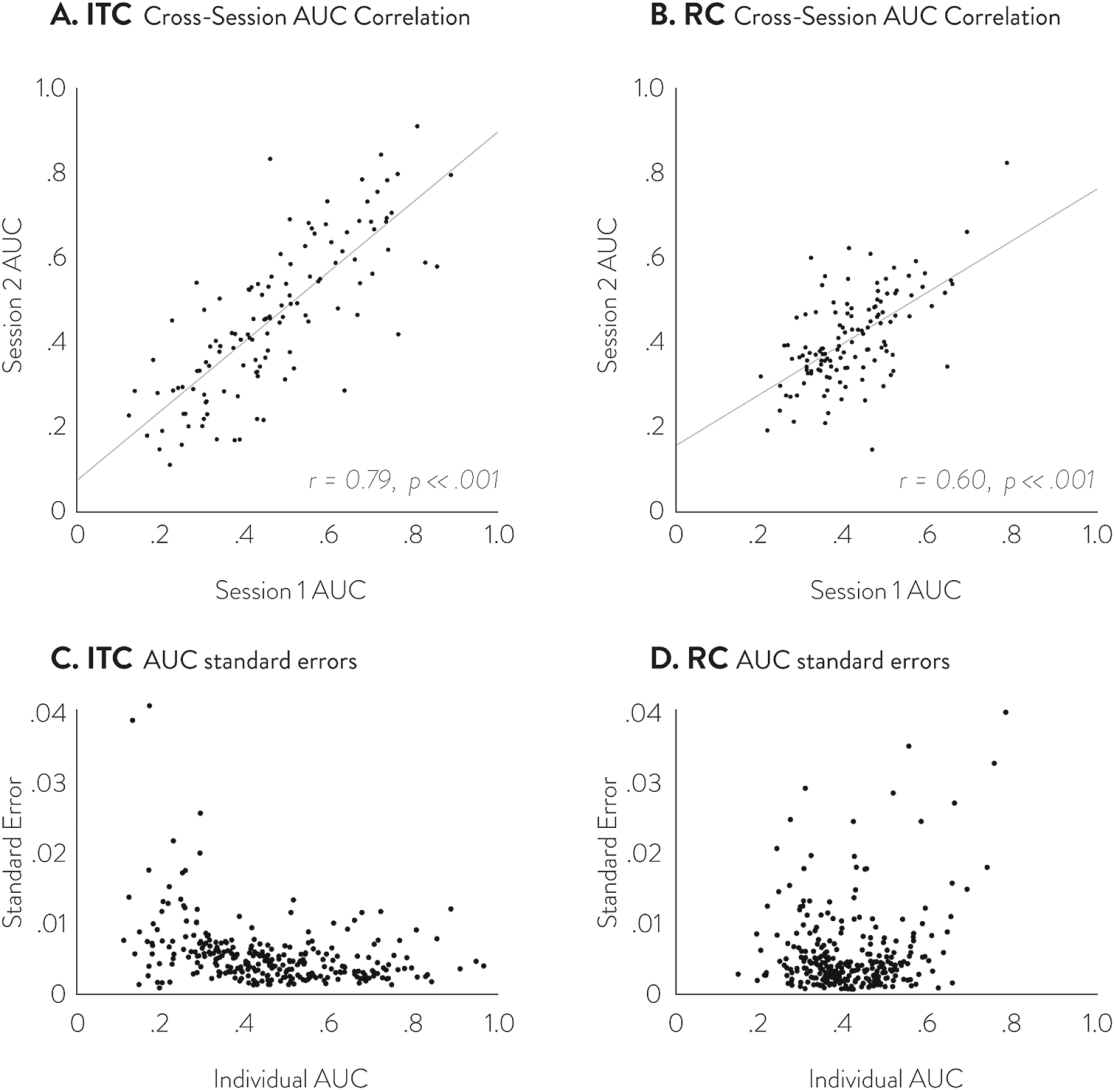
Cross-session correlations and standard errors of overall measures of delay discounting (**a**, **c**) and risk aversion (**b**, **d**) as estimated by the Area Under the Curve (AUC) of CBS. In **a**, **b**, the abscissa marks the AUC measure of each participant in session 1 and the ordinate marks the AUC measure of each participant in session 2. The cross-session Pearson correlation measure of AUC was 0.79 for ITC and 0.60 for RC, both with p-values less than .001. **c**, **d** shows the standard errors of the AUC estimates obtained through a jackknife procedure

### Identifying Novel Patterns of Behavior

To provide further insight into why CBS shows increased predictive power over parametric models, we first present example participants’ data that span the variety of choice patterns we observe. Example participants’ choices and model fits are shown for ITC in Fig. 6 and for RC in Fig. 7. Both the choices and fits are shown in relative amounts. The relative amount is the immediate amount divided by the larger amount; for example, in ITC, a choice of $20 versus $40 in 6 days is essentially asking if $$f(D = 6$$) is greater or less than 0.5, which is the relative amount of 20/40. By plotting each question in terms of relative amount and delay, we can see whether the fitted function (drawn in solid black line) is appropriately dividing the two choice types (shown in circles and Xs). Panel A in both Figures shows 4 participants’ data whose highest LOOCV Tjur’s D is from extant parametric models. The top row shows the parametric model fits while the middle row shows the CBS model fits. In these cases, participants’ choices are well aligned with known parametric models and CBS shows good approximations of them. Given larger datasets, CBS will likely match the parametric models in these participants. On the other hand, Panel B in both Figures shows 4 participants’ data whose highest LOOCV Tjur’s D is from CBS. We can see in the top row that even the best extant parametric models are unable to separate the choices well. In contrast, CBS fits a rather unconventional, but flexible, monotonic function that separates the two choice types.

More specifically, participants who are best fit by CBS in Figs. 6 and 7 seem to exhibit choice patterns that cannot be accounted for by parametric models. Figure 6 shows that in ITC, several participants exhibit a discounting function that decreases sharply at certain delays. Such sharp decreases in utility indicate suddenly increasing impatience and discount rates, which cannot be accounted for by any of the parametric models we considered. Figure 7 shows that in RC, several participants exhibit a complex discounting function with multiple inflection points. Generally, these participants are risk-averse in low probabilities (as shown by the fitted curve being below the identity line), risk-seeking around $p = .5$, and risk-averse again above .5. This pattern of multiple switches between risk-aversion and risk-seeking behavior deviates from the established parametric models which can only account for either overall risk-aversion or risk-seeking throughout all probabilities, or a one-time switch between risk-aversion and risk-seeking.

Group-level summaries confirm that the predictive advantages of CBS are largest when participants exhibit these novel systematic patterns of choice behavior. In ITC, the novel choice pattern is that many participants have increasing daily discount rates, which is inconsistent with all the parametric models we examine. When we group the fitted CBS functions based on the average daily change in discount rate, the best parametric model’s LOOCV Tjur’s D is as good as that of CBS models when participants have decreasing discount rates, which is the commonly assumed pattern. However, when the average daily discount rates are increasing, the CBS models significantly outperform the best parametric models in LOOCV (Fig. 8a). When we examine the fitted CBS functions, we find that when the average daily change in discount rate is negative, the median CBS function looks very similar to other ITC parametric models; on the other hand, when the average daily change in discount rate is positive (i.e., increasing discount rates over time), we find that the median CBS function becomes linear or even concave, neither of which could be accounted for by parametric ITC models (Fig. 8b).

In RC, the novel choice pattern is that many participants switch multiple times between risk-aversion and risk-seeking as probabilities increase, which is inconsistent with all the parametric models we examine. Concordantly, CBS’s LOOCV Tjur’s D are significantly higher than the best parametric models’ LOOCV Tjur’s D for participants with 2 or more switches (Fig. 8c). This result suggests that participants exhibit potentially much more complex patterns of behavior than what most parametric models assume. Interestingly, even in participants that do not switch between risk-aversion and risk-seeking, we find that CBS significantly outperforms other parametric models in LOOCV. Figure 8d shows the median CBS-fitted functions grouped by the number of switches between risk-averse and risk-seeking behavior (as seen by how many times the function crosses the identity line). When participants switch once, their average function resembles a typical prospect theory S-shaped function (albeit risk-averse in low probabilities). This simple form is likely captured well by most parametric utility models, thereby leading to similar predictive performance between parametric and CBS models. However, when participants’ risk aversion switches twice or three times, the average function clearly cannot be captured by any of the parametric RC models. Furthermore, although the parametric utility models can account for non-switching behavior as well, the average function for non-switching behavior has some inflection points that cannot be captured by the parametric models (cf. Fig. 2b).

### Model-Agnostic Measures of Impulsivity and Risk Aversion

We find the CBS measures of impulsivity and risk-aversion are highly correlated across the 2 sessions, 10 weeks apart (Fig. 9). This result suggests that CBS measures of impulsivity and risk-aversion can pick up stable individual traits that are often needed in applied ITC and RC research. Using the 2-piece CBS fits to the real choice data, we calculate, for each session, an overall measure of impulsivity and risk aversion by calculating the AUC of the fitted CBS function. The cross-session Pearson correlations of the AUCs are very high at $r = 0.79$ ($p < .001$) for ITC and $r = 0.60$ ($p< .001$) for RC. These measures are comparable to the cross-session consistencies of extant parametric models’ impulsivity and risk aversion measures; the hyperbolic model’s discount rate (logk) has a cross-session correlation of $r = 0.80$, and EUT’s risk-aversion measure (log $\alpha )$ has a cross-session correlation of $r= 0.65$. Furthermore, the standard error of the AUC measures in CBS is quite low (generally below .01); higher standard errors are observed, expectedly, from cases where participants’ choices are heavily one-sided and do not allow for good measurement (i.e., extreme ends of AUC measures on Fig. 9c, d). This shows that CBS fits can yield a stable individual-specific measure of overall impulsivity or risk aversion without assuming a fully parametric model.

## Discussion

Cubic Bezier Splines are a promising flexible method that can approximate individual utility functions without fully parametric assumptions. As a structured non-parametric method, it maintains the interpretable utility function structure found in parametric models but relaxes the parametric assumptions, thereby increasing descriptive and predictive capabilities. Unlike previous structured non-parametric approaches however (e.g., Abdellaoui 2003; Myerson et al. 2006; Wakker and Deneffe 1996), CBS can be estimated from any choice dataset without large or specially structured datasets. Such properties allow us to demonstrate both the predictive and interpretive advantages of CBS modeling in a general ITC and RC dataset that was not specifically designed toward non-parametric estimation.

In prediction, we show that CBS can provide higher descriptive and predictive performance compared to extant parametric models. Through simulation, we show that the benefit of a flexible CBS approximation outweighs the benefit of parsimonious parametric models even in small datasets of around 50 binary choices. Hence, unlike fully non-parametric approaches which seem to require large datasets (Arfer and Luhmann 2015), a CBS-based structured non-parametric approach does not seem to require substantially larger datasets than what would be normally used for parametric model estimation. We empirically validate this result by showing that in a real dataset of 120 choices, CBS shows higher in-sample and out-of-sample predictive performance compared to all tested parametric models. This is likely due to CBS’s ability to provide individually tailored utility functions, which lead to improved descriptive (in-sample) and predictive (out-of-sample) capabilities. In datasets with heterogeneous utility functions, having an individually tailored utility function allows researchers to circumvent potential model misspecifications. Using CBS approximations provides a stronger defense against model misspecifications than entertaining a multitude of models as empirical data may not be describable by any known parametric models.

CBS, as a structured non-parametric approach, also yields interpretable insights and measures from data that cannot be obtained as easily from fully non-parametric models. First, CBS can be used to detect novel patterns of behavior that violate extant models’ assumptions. In the current paper, we identify two novel patterns of behavior from ITC and RC data. In ITC, we find that there are participants who exhibit increasing discount rates and therefore cannot be accounted for by the currently established parametric models of ITC. Such participants exhibit concave utility functions which may be indicative of a heuristic (e.g., deciding not to wait after a certain delay). In RC, we find that there are participants who alternate between risk-aversion and risk-seeking multiple times within the probability range of [0 1]. Such complex patterns of behavior cannot be described by the established parametric models of RC which assume at most one switch between risk-aversion and risk-seeking behavior. Future studies in ITC and RC may be able to identify new ways of clustering these kinds of patterns to identify participants who may use different sets of psychological processes when making decisions (e.g., Reeck et al. 2017). Second, CBS provides measures of impatience and risk aversion that do not depend on a specific parametric utility model. Given the heterogeneity of utility functions in choice data, there has always been a need to characterize individual’s overall behavior without having to rely on a specific model (Myerson et al. 2006). The area under the curve (AUC) of the estimated CBS function serves as an overall measure of impulsivity or risk aversion that is robust to model misspecifications even in the face of heterogeneous data.

CBS also has the potential to aid other research questions, some of which we list here. First, it can aid the study of choice stochasticity by more accurately dissociating between model misspecification and choice noise. Goodness-of-fit measures for parametric utility functions do not provide good assessments of choice noise because one cannot distinguish whether the data is stochastically noisy or if the utility model is simply misspecified. Previous research has focused on the monotonicity of utility functions to make a theoretical distinction between model misspecification and genuine noise (Johnson and Bickel 2008). Since the CBS models that we present here have only the general normative assumption of monotonicity, the noise estimates from CBS only includes the stochasticity that cannot be explained with a monotonic utility function. Future research may seek to correlate choice stochasticity with other measures such as impulsivity, risk-aversion, age, education, and/or IQ.

Second, CBS can provide more accurate estimates of latent utilities, which will also aid current efforts to relate such utilities to other behavioral and neural measures (Levy and Glimcher 2012; Venkatraman et al. 2014). For example, numerous studies have examined drift-diffusion and similar models that can incorporate both response time and choice data (Busemeyer and Townsend 1993; Clithero 2018; Dai and Busemeyer 2014; Forstmann et al. 2016; Ratcliff et al. 2016). By using utility estimates that can describe participants’ choices better than traditional parametric utility estimates, development and validation of these models can be improved. Also, decision neuroscience research often requires estimates of utilities that can be used to search for correlates of valuation in the brain (e.g., Kable and Glimcher 2007; Knutson 2005). These efforts can also benefit from more refined estimates of utility that better predicts participants’ choices.

Despite these substantial benefits, it is important to note that there are some drawbacks of flexible approaches like CBS. CBS, or at least the current version, is best used on datasets that have reasonable coverage over a range of values, as there is no ‘default’ shape that CBS tends toward in absence of data. In future research, CBS’s extrapolation capabilities can be enhanced by using priors or penalties toward a commonly used utility function (e.g., hyperbolic, or EUT) such that CBS can default to more simple forms in the absence of data, but take on a more complex form given sufficient data. Furthermore, while the form of CBS function used in this paper can approximate a large number of extant parametric models, there are several models that cannot be fully approximated by CBS such as attribute comparison models (Dai and Busemeyer 2014) and mean-variance models of risky choice (Markowitz 1959; Weber et al. 2004).

As we provide CBS as a new tool for describing, understanding, and predicting decisions, we hope that this research is the start of using flexible models to explore many topics not only related to economic decision-making, but also other cognitive, affective, and social behaviors whose models have latent variables. We hope that across many areas of human behavior, the behavioral patterns and heterogeneity that went unnoticed under formal parametric assumptions can now easily be brought to surface and studied.

## Electronic supplementary material

Below is the link to the electronic supplementary material.

Supplementary material 1 (pdf 751 KB)
