# Supplementary material for: Flexible Utility Function Approximation via Cubic Bezier Splines
Source: Psychometrika. 2020 Sep 26;85(3):716–37. doi: 10.1007/s11336-020-09723-4 (PMC7599200; doi:10.1007/s11336-020-09723-4)
Supplement: Supplementary file 1 — Supplementary material 1 (pdf 751 KB) [file 11336_2020_9723_MOESM1_ESM.pdf]

A. Transformation of models with amount transformation functions

In most cases, utility functions of the form $U = f(A) \cdot g(X)$ can be simplified to a discounting form. This is particularly desirable given that two simultaneous variable transformations are unidentifiable in most choice datasets. In the case of prospect theory $U = f(A) \cdot g(p)$, if one's $f(A)$ is generally high, one can lower the $g(p)$ to compensate to achieve the same utility. Fox and Poldrack (2009) noted this difficulty and suggested that researchers take caution. Bruhin et al. (2009) have noted in their paper that "fitting a (prospect theory) model for each individual is … frequently impossible and often not desirable in the first place."

A relationship between two options' utilities $f(A_1) \cdot g(X_1) \gtreqless f(A_2) \cdot g(X_2)$ can be analytically converted to a discounting form if the inverse function of the amount transformation f has a monotonically increasing and distributive property : $A_1 \cdot f^{-1}\big(g(X_1)\big) \gtreqless A_2 \cdot f^{-1}\big(g(X_2)\big)$. Given that $f(A)$ is a amount function, it is expected to be monotonically increasing. On the other hand, not all proposed amount function's inverses have a distributive property. However, the most commonly used amount function $f(A) = A^{\alpha}$ does have a distributive property such that $(AB)^{1/\alpha} = A^{1/\alpha}B^{1/\alpha}$. For example, consider a simple gamble and its certainty equivalent: $CE^{\alpha} = A^{\alpha} \cdot wp$. We can de-exponentiate both sides to achieve $CE = A \cdot wp^{1/\alpha}$. Since wp is being raised to an exponent, it does not alter its values at 0 and 1 which still remain 0 and 1 after exponentiation. Hence this can be estimated generally using the form $U = A \cdot f(p)$ with CBS.

B. Monotonic B-splines and relationship to CBS

Basis-splines (more commonly referred to as B-splines), which CBS is a special case of, have been used in scenarios with monotonicity constraints (Brezger & Steiner, 2008; Leitenstorfer & Tutz, 2007); but the monotonicity constraint of B-splines has only been worked out for evenly spaced knots, which does not allow the data to determine the appropriate position of the knots. In this paper, we provide a method for allowing the data to control the position of the knots, thereby providing a more flexible approach with a fewer number of parameters (knots). This is done by connecting multiple small B-splines, each of which only has four knots, and can analytically be constrained for monotonicity even with free positioning of the knots. Here we use Bernstein basis function for the splines which makes them Bezier splines.

C. Compulsory and Slope Conditions of CBS

Because chains of CBS are locally adjustable, the derivation of constraints only need to be worked out with regards to one CBS. A single piece of CBS is described by the following two parametric curves:

$$x(t) = (1-t)^3 x_1 + 3(1-t)^2 t x_2 + 3(1-t)t^2 x_3 + t^3 x_4, \qquad 0 \le t \le 1 \tag{1}$$

$$y(t) = (1-t)^3 y_1 + 3(1-t)^2 t y_2 + 3(1-t)t^2 y_3 + t^3 y_4, \qquad 0 \le t \le 1 \tag{2}$$

In order to use CBS to approximate a function of the form $y = f(x)$, we can assume $x_1 < x_4$ in order to have a spline of non-zero length. Also, we must ensure that $x(t)$ is a monotonically increasing function of t in $[0 \;\; 1]$. Otherwise, multiple values of y may exist for one x.

One may also want to impose additional constraints on CBS. In terms of the first derivative, one can make it monotonically increasing or monotonically decreasing. Given that $x(t)$ is a monotonically increasing function of t, it is only necessary to control for $y(t)$ for this slope constraint. One may also want to constrain CBS with the sign of the second derivative to be concave or convex.

The derivative of $x(t)$ with regards to t is as follows:

$$\frac{dx}{dt} = 3\{(-x_1 + 3x_2 - 3x_3 + x_4)t^2 + 2(x_1 - 2x_2 + x_3)t - x_1 + x_2\} \tag{3}$$

To ensure that dx/dt is positive in [0 1]: 1) dx/dt is positive at $t = 0$ and $t = 1$, and 2) $dx/dt = 0$ has no real roots in [0 1]. The first condition gives the following two inequalities:

$$x_1 < x_2, \qquad x_3 < x_4 \tag{4}$$

For the second condition, we employ a monotonic transformation of $z = t/(1-t)$, in which case $0 < t < 1$ translates to $0 < z$. Then we only need to ensure that $dx/dz = 0$ does not have any positive real roots. After conversion and arrangement, $dx/dz = 0$ becomes the following:

$$(x_3 - x_4)z^2 + 2(x_2 - x_3)z + x_1 - x_2 = 0 \tag{5}$$

Since $x_3 \neq x_4$, equation (5) is quadratic and the roots are the following:

$$z = \frac{(x_3 - x_2) \pm \sqrt{(x_3 - x_2)^2 - (x_2 - x_1)(x_4 - x_3)}}{x_3 - x_4} \tag{6}$$

It must be that either the determinant is negative or that it is non-negative but the roots are negative. In order for the determinant to be negative, the following must be true:

$$-\sqrt{(x_4 - x_3)(x_2 - x_1)} < x_3 - x_2 < \sqrt{(x_4 - x_3)(x_2 - x_1)} \tag{7}$$

If the determinant is non-negative, the converse is true:

$$x_3 - x_2 \leq -\sqrt{(x_4 - x_3)(x_2 - x_1)} \qquad or \qquad \sqrt{(x_4 - x_3)(x_2 - x_1)} \leq x_3 - x_2 \tag{8}$$

If the left part of inequality (8) is true, it means that $x_3 - x_2$ is negative, which leads to at least one root of z (equation 6) being positive (since the denominator is negative). Hence, only the right part of inequality (8) can be true.

Combining all our results so far, we have the following compulsory conditions:

$$x_1 < x_4, \qquad x_1 < x_2, \qquad x_3 < x_4, \qquad -\sqrt{(x_4 - x_3)(x_2 - x_1)} < x_3 - x_2 \tag{9}$$

While these conditions guarantee that $x(t)$ is a monotonic function of t, we found that it is not ideal in that the final constraint is a non-linear inequality that entangles the 4 coordinates simultaneously, thereby reducing their independence. For this reason, we provide a more restrictive constraint of monotonicity that also allows the parameters to be more independent from each other:

$$x_1 < x_4, \qquad x_1 < x_2, \qquad x_3 < x_4, \qquad x_1 < x_3, \qquad x_2 < x_4 \tag{10}$$

These constraints make it so that as long as x_2 and x_3 stay within $[x_1, x_4]$, monotonicity is conserved. We can show that this satisfies the conditions on (9) by the following proof.

If $x_2 < x_3$, then $0 < x_3 - x_2$, which obviously satisfies $-\sqrt{(x_4 - x_3)(x_2 - x_1)} < x_3 - x_2$. If otherwise $(x_2 \geq x_3)$, then it means that $x_1 < x_3 \leq x_2 < x_4$ under conditions in (10). Then, we can see that $(x_4 - x_3)(x_2 - x_1) = \{(x_4 - x_2) + (x_2 - x_3)\}\{(x_2 - x_3) + (x_3 - x_1)\} = (x_2 - x_3)^2 +$

$(x_4 - x_2)(x_2 - x_3) + (x_3 - x_1)(x_2 - x_3) + (x_4 - x_2)(x_3 - x_1)$ and that this is strictly greater $(x_3 - x_2)^2$ since all terms are positive. Therefore, $-\sqrt{(x_4 - x_3)(x_2 - x_1)} < x_3 - x_2$ is satisfied.

Finally, we can see that the very first inequality in (10) is now obsolete because the other inequalities (e.g., $x_1 < x_2$, $x_2 < x_4$) already imply it. The constraint for slope is very similar to the compulsory condition as one only needs to swap x and y.

D. Curvature Conditions of CBS

Constraint for curvature must be done using the second derivative d^2y/dx^2:

$$\frac{d^2y}{dx^2} = \frac{\frac{d}{dt}\left(\frac{dy/dt}{dx/dt}\right)}{\frac{dx}{dt}} = \frac{\frac{d^2y}{dt^2}\frac{dx}{dt} - \frac{dy}{dt}\frac{d^2x}{dt^2}}{\left(\frac{dx}{dt}\right)^3} \quad (11)$$

Since our interest lies in constraining the sign of the 2nd derivative, the denominator is unnecessary for our purpose as it is always positive. The numerator becomes the following quadratic function of t:

$$pt^2 + qt + r \quad (12)$$

$$p = (y_1 - 2y_2 + y_3)(x_1 - 3x_2 + 3x_3 - x_4) - (x_1 - 2x_2 + x_3)(y_1 - 3y_2 + 3y_3 - y_4)$$

$$q = (x_1 - x_2)(y_1 - 3y_2 + 3y_3 - y_4) - (y_1 - y_2)(x_1 - 3x_2 + 3x_3 - x_4)$$

$$r = (y_1 - y_2)(x_1 - 2x_2 + x_3) - (x_1 - x_2)(y_1 - 2y_2 + y_3)$$

Let V_0 and V_1 denote the evaluation of equation 11 at $t = 0$ and $t = 1$:

$$\left.\frac{d^2y}{dx^2}\right|_{t=0} \propto r = V_0 = -x_2y_1 + x_3y_1 + x_1y_2 - x_3y_2 - x_1y_3 + x_2y_3 \quad (13)$$

$$\left.\frac{d^2y}{dx^2}\right|_{t=1} \propto p + q + r = V_1 = -x_3y_2 + x_4y_2 + x_2y_3 - x_4y_3 - x_2y_4 + x_3y_4 \quad (14)$$

The interpretation of V_0 and V_1 becomes clear when one considers their various forms:

$$\begin{aligned} V_0 &= -x_2y_1 + x_3y_1 + x_1y_2 - x_3y_2 - x_1y_3 + x_2y_3 \\ &= (x_2 - x_1)(y_3 - y_1) - (x_3 - x_1)(y_2 - y_1) \\ &= (x_2 - x_3)(y_3 - y_1) - (x_3 - x_1)(y_2 - y_3) \\ &= (x_2 - x_3)(y_2 - y_1) - (x_2 - x_1)(y_2 - y_3) \end{aligned} \quad (15)$$

$$\begin{aligned} V_1 &= -x_3y_2 + x_4y_2 + x_2y_3 - x_4y_3 - x_2y_4 + x_3y_4 \\ &= (x_4 - x_2)(y_4 - y_3) - (x_4 - x_3)(y_4 - y_2) \\ &= (x_4 - x_2)(y_2 - y_3) - (x_2 - x_3)(y_4 - y_2) \\ &= (x_4 - x_3)(y_2 - y_3) - (x_2 - x_3)(y_4 - y_3) \end{aligned} \quad (16)$$

As can be seen from above, a constraint of $V_0 > 0$ or $V_1 < 0$ is essentially constraining the relationship between the slopes between the three points (points 1,2, and 3 for V_0, and points 2,3, and 4 for V_1).

In order for the entire CBS to convex, it does not suffice for the signs of V_0 and V_1 to be both positive. We must additionally ensure that equation 11 does not have a root between 0 and 1. Again, we use a monotonic transformation$z = t/(1-t)$, in which case the relevant part (the part that modulates the sign) of equation 11 becomes the following: $V_1 z^2 + (q+2r)z + V_0$. Then, the two roots of this formula is as follows:

$$\frac{-(q+2r) \pm \sqrt{(q+2r)^2 - 4V_0V_1}}{2V_1} \tag{17}$$

If the determinant is negative, it means the following constraint is true:

$$-2\sqrt{V_0V_1} < q + 2r < 2\sqrt{V_0V_1} \tag{18}$$

If the determinant is non-negative, it means the converse is true:

$$q + 2r \leq -2\sqrt{V_0V_1} \quad or \quad 2\sqrt{V_0V_1} \leq q + 2r \tag{19}$$

Since $V_1 > 0$, we know that the denominator of equation 16 is positive and hence the numerator must be negative. If the left part of inequality 18 is true, it follows that both roots of equation 16 is positive. Hence only the right side of inequality 18 can hold. Combining the constraints, we have the following for a fully convex curve:

$$V_0 > 0, \qquad V_1 > 0, \qquad -2\sqrt{V_0V_1} < q + 2r \tag{20}$$

While this is a sufficient condition of a fully convex curve, it is difficult to utilize because the underlying variables are intertwined. If we use the monotonicity constraint from the compulsory condition (9), we can simplify this (20) further.

First, we prove that $V_0 > 0, V_1 > 0, 0 < q + 2r$ is a sufficient condition for convexity using proof by contradiction. Let's assume that there is a fully convex curve that satisfies the following constraints: $V_0 > 0, V_1 > 0, -2\sqrt{V_0V_1} < q + 2r \leq 0$.

If$x_2 < x_3$, it implies $x_1 < x_2 < x_3 < x_4$ by (9), and the following conditions hold:

$$V_0 > 0 \Leftrightarrow (x_2 - x_1)(y_3 - y_1) - (x_3 - x_1)(y_2 - y_1) > 0 \Leftrightarrow \frac{y_3 - y_1}{x_3 - x_1} > \frac{y_2 - y_1}{x_2 - x_1} \tag{21}$$

$$V_1 > 0 \Leftrightarrow (x_4 - x_2)(y_4 - y_3) - (x_4 - x_3)(y_4 - y_2) > 0 \Leftrightarrow \frac{y_4 - y_3}{x_4 - x_3} > \frac{y_4 - y_2}{x_4 - x_2} \tag{22}$$

$$q + 2r \leq 0 \Leftrightarrow (x_2 - x_1)(y_4 - y_3) - (x_4 - x_3)(y_2 - y_1) \leq 0 \Leftrightarrow \frac{y_4 - y_3}{x_4 - x_3} \leq \frac{y_2 - y_1}{x_2 - x_1} \tag{23}$$

Also, by the inequality of arithmetic and geometric means, we also see that if $-2\sqrt{V_0V_1} < q + 2r$ holds, $-(V_0 + V_1) < q + 2r$ also holds, giving us the following inequality:

$$-(V_0 + V_1) < q + 2r \Leftrightarrow (x_3 - x_1)(y_4 - y_2) - (x_4 - x_2)(y_3 - y_1) > 0 \Leftrightarrow \frac{y_4 - y_2}{x_4 - x_2} > \frac{y_3 - y_1}{x_3 - x_1} \tag{24}$$

Combination of inequality 21, 22, and 24 give us the following inequality:

$$\frac{y_4 - y_3}{x_4 - x_3} > \frac{y_4 - y_2}{x_4 - x_2} > \frac{y_3 - y_1}{x_3 - x_1} > \frac{y_2 - y_1}{x_2 - x_1} \tag{25}$$

However, this is directly contradicted by inequality 23. Hence $x_2 < x_3$ cannot hold.

If $x_2 \geq x_3$, we expand the condition of $-2\sqrt{V_0V_1} < q + 2r \leq 0$:

$$-2\sqrt{\left((x_2-x_3)(y_2-y_1)-(x_2-x_1)(y_2-y_3)\right)\left((x_4-x_3)(y_2-y_3)-(x_2-x_3)(y_4-y_3)\right)} < (x_2-x_1)(y_4-y_3)-(x_4-x_3)(y_2-y_1) \leq 0 \quad (26)$$

From the compulsory condition on equation 9, we have $\sqrt{(x_4-x_3)(x_2-x_1)} > x_2 - x_3$. We can multiply both sides by $\sqrt{(x_4-x_3)(x_2-x_1)}$, which gives us $(x_4-x_3)(x_2-x_1) > (x_2-x_3)\sqrt{(x_4-x_3)(x_2-x_1)}$. Since both sides of inequality 26 are negative, we can divide the left side with a smaller positive number and the right side with a larger positive number and still maintain the inequality. Hence we divide the left side with $(x_2-x_3)\sqrt{(x_4-x_3)(x_2-x_1)}$ and the right side with $(x_4-x_3)(x_2-x_1)$. This gives us the following:

$$-2\sqrt{\left(\frac{y_2-y_1}{x_2-x_1}-\frac{y_2-y_3}{x_2-x_3}\right)\left(\frac{y_2-y_3}{x_2-x_3}-\frac{y_4-y_3}{x_4-x_3}\right)} < \frac{y_4-y_3}{x_4-x_3}-\frac{y_2-y_1}{x_2-x_1} \quad (27)$$

Applying the inequality of arithmetic and geometric mean on the left side, we have the following:

$$-\left(\frac{y_2-y_1}{x_2-x_1}-\frac{y_2-y_3}{x_2-x_3}+\frac{y_2-y_3}{x_2-x_3}-\frac{y_4-y_3}{x_4-x_3}\right) < \frac{y_4-y_3}{x_4-x_3}-\frac{y_2-y_1}{x_2-x_1} \quad (28)$$

However, the left-side equals the right side and the inequality provides a contradiction. Hence whether $x_2 < x_3$ or $x_2 \geq x_3$, our initial assumption of $V_0 > 0$, $V_1 > 0$, $-2\sqrt{V_0V_1} < q + 2r \leq 0$ does not hold. Concordantly, we have $V_0 > 0, V_1 > 0, 0 < q + 2r$.

Now if we assume $x_3 \leq x_2$, these conditions give us contradiction:

$$V_0 > 0, V_1 > 0 \Leftrightarrow \frac{y_4-y_3}{x_4-x_3} < \frac{y_3-y_2}{x_3-x_2} < \frac{y_2-y_1}{x_2-x_1} \quad (29)$$

$$q + 2r > 0 \Leftrightarrow \frac{y_4-y_3}{x_4-x_3} > \frac{y_2-y_1}{x_2-x_1} \quad (30)$$

Hence, $x_2 < x_3$ and the convexity condition can be simplified as the following:

$$\frac{y_2-y_1}{x_2-x_1} < \frac{y_3-y_2}{x_3-x_2} < \frac{y_4-y_3}{x_4-x_3} \quad (31)$$

E. Smoothness Conditions of multi-piece CBS

So far, all the constraints have been worked out with regards to a single piece of CBS. However, in order to model more complex functions, one would need to chain multiple pieces of CBS functions together. In order to guarantee a smooth transition between the two chained CBS functions, there are some constraints that one should impose.

First, the derivative of CBS must be continuous at the joining point of two CBS functions. This is straightforward as the line between the anchor point and its control point marks the local derivative. Hence, one should just ensure that the two control points of an anchor point is on the same line. Second, if the control handles (i.e., the distance between anchor point and its control points) becomes very short, there is potential for a kink in that location (**Supplemental Fig 1**). Therefore, it can be useful to constrain the minimal distance between control points and their anchor point.

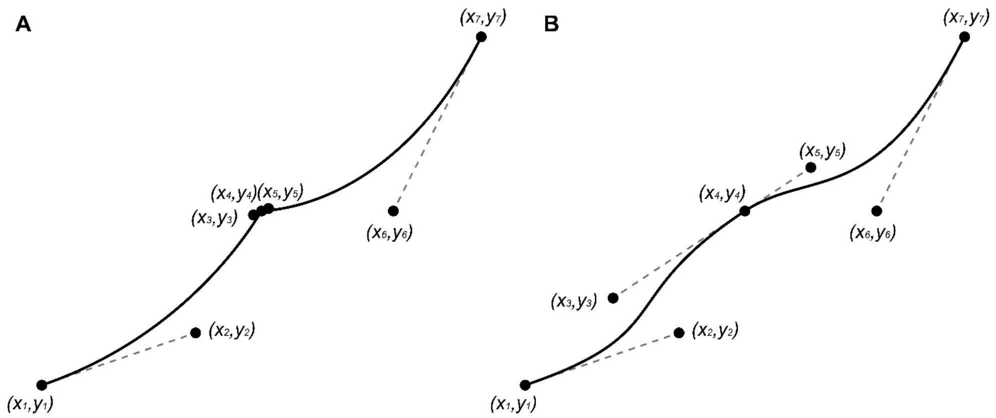

Supplemental Figure 1. Two-piece CBS. *Panel A on the left shows the kink that forms when the control points become too close to the anchor point at the joining point of two CBS functions. Panel B shows a smooth transition between two pieces of CBS functions thanks to the appropriate distance between control points and anchor point.*

The table below summarizes all the constraints.

<table>
<tr><td>Compulsory</td><td colspan="2">$x_1 < x_2 < x_4, \quad x_1 < x_3 < x_4$</td></tr>
<tr><td rowspan="2">Slope</td><td>Monotonically increasing</td><td>$y_1 < y_2 < y_4, \quad y_1 < y_3 < y_4$</td></tr>
<tr><td>Monotonically decreasing</td><td>$y_1 > y_2 > y_4, \quad y_1 > y_3 > y_4$</td></tr>
<tr><td rowspan="2">Curvature</td><td>Convex</td><td>$\frac{y_2 - y_1}{x_2 - x_1} < \frac{y_3 - y_2}{x_3 - x_2} < \frac{y_4 - y_3}{x_4 - x_3}$</td></tr>
<tr><td>Concave</td><td>$\frac{y_2 - y_1}{x_2 - x_1} > \frac{y_3 - y_2}{x_3 - x_2} > \frac{y_4 - y_3}{x_4 - x_3}$</td></tr>
<tr><td>Smoothness</td><td colspan="2">For a n – piece CBS function,
$\frac{y_i - y_{i-1}}{x_i - x_{i-1}} = \frac{y_{i+1} - y_i}{x_{i+1} - x_i}, \quad \forall i = 3j + 1, \quad j \in \{1,2,\ldots,n-1\}.$
And,
$m^2 < (x_i - x_{i-1})^2 + (y_i - y_{i-1})^2, \quad m^2 < (x_{i+1} - x_i)^2 + (y_{i+1} - y_i)^2$
For a small number m (m = 0.1 in this paper).</td></tr>
</table>

F. Summary measure of impulsivity and risk-aversion via CBS AUC

The area under the curve (AUC) of fitted CBS functions serves as a summary measure of impulsivity and risk-aversion. In the case of a 2-piece CBS function, one can add up the AUC of each individual piece. The formula for AUC can be calculated quite straightforwardly by applying integration on equations (1) and (2). Let (x_i, y_i) denote the coordinates of the point P_i. Then, the AUC of each CBS piece is as follows:

$$\int_{x_0}^{x_3} y\,dx = \frac{1}{20}\begin{pmatrix} 6x_1y_0 - 6x_0y_1 - 10x_0y_0 - 3x_0y_2 + 3x_2y_0 - x_0y_3 - 3x_1y_2 \\ +3_{x_2}y_1 + x_3y_0 - 3x_1y_3 + 3x_3y_1 - 6x_2y_3 + 6x_3y_2 + 10x_3y_3 \end{pmatrix} \tag{32}$$

G. Simulated Utility Function Recovery Using CBS

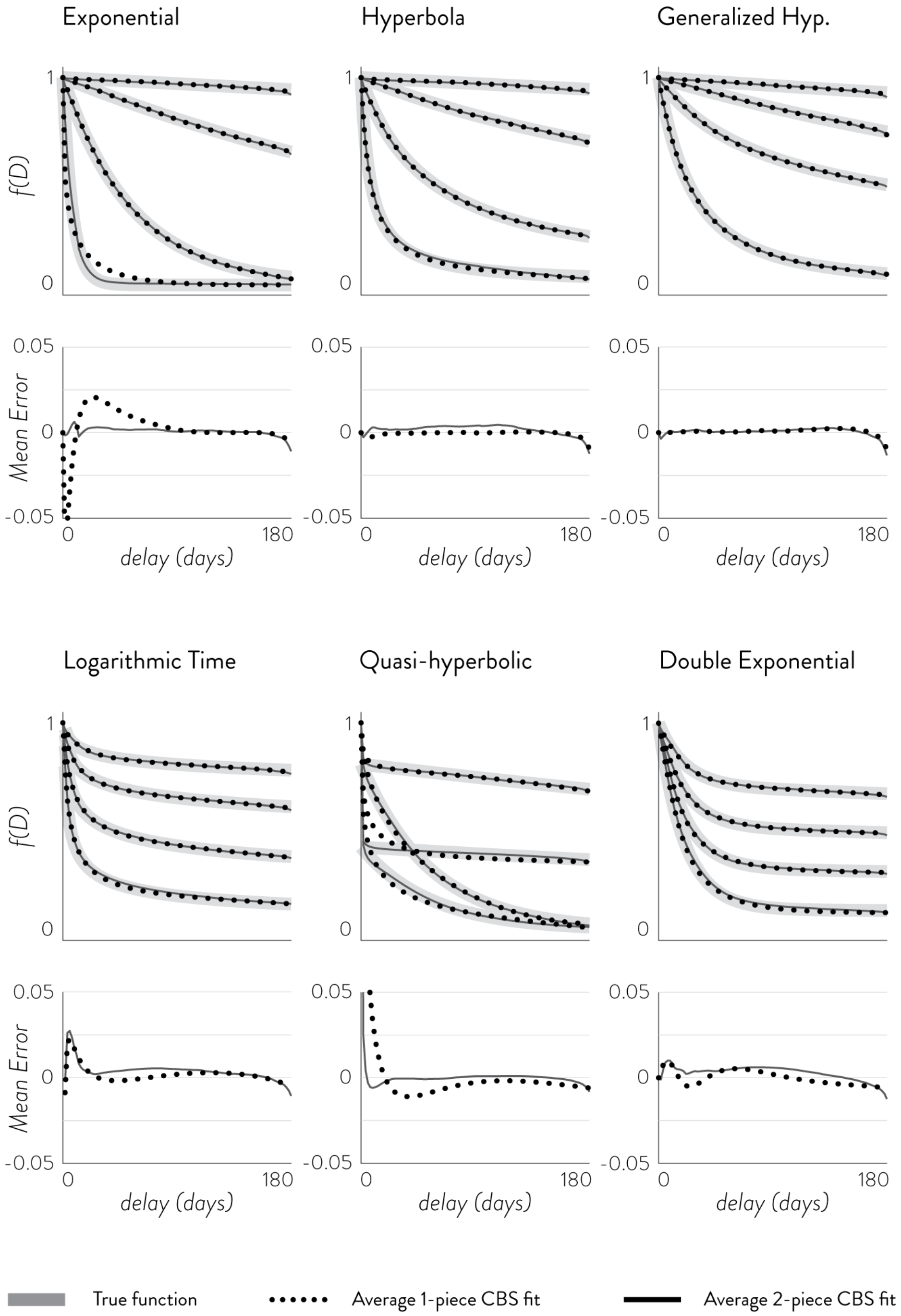

Supplemental Figure 2. Average CBS fits from ITC choice dataset simulation. *CBS fits are shown overlaid on top of 6 different ITC utility functions. The first and the third row shows the true simulating utility functions and their average CBS fits. The average CBS fits were calculated by taking the mean of the 200 fitted CBS functions from the largest choice dataset (400 choices). The second and the fourth row shows the mean error of the fitted CBS functions and the true simulating functions.*

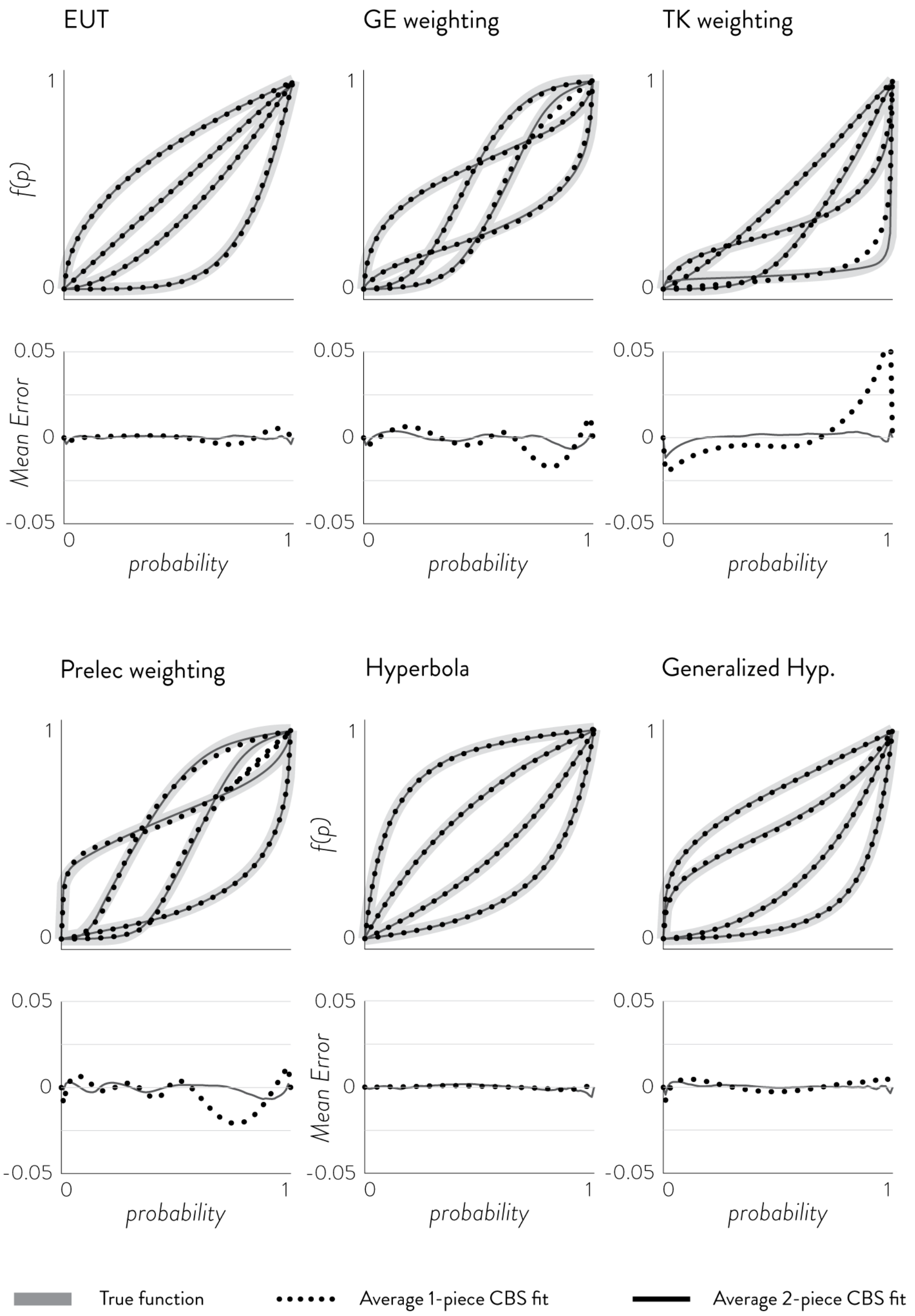

Supplemental Figure 3. Average CBS fits from RC dataset simulation. *CBS fits are shown overlaid on top of 6 different RC utility functions (converted into probability space). The first and the third rows show the true simulating utility functions and their average CBS fits. The average CBS fits were calculated by taking the mean of the 200 fitted CBS functions from the largest choice dataset (400 choices). The second and the fourth rows show the mean error of the fitted CBS functions and the true simulating functions.*

H. Supplemental references

Brezger, A., & Steiner, W. J. (2008). Monotonic Regression Based on Bayesian P–Splines. *Journal of Business & Economic Statistics*. https://doi.org/10.1198/073500107000000223

Bruhin, A., Fehr-Duda, H., & Epper, T. (2009). Risk and Rationality: Uncovering Heterogeneity in Probability Distortion. *Ssrn*, *78*(4), 1375–1412.

Fox, C. R., & Poldrack, R. A. (2009). Prospect theory and the brain. In *Neuroeconomics* (pp. 145–173).

Leitenstorfer, F., & Tutz, G. (2007). Generalized monotonic regression based on B-splines with an application to air pollution data. *Biostatistics*. https://doi.org/10.1093/biostatistics/kxl036
